# Face mask ownership/utilisation and COVID-19 vaccine hesitancy amongst patients recovering from COVID-19 in Cameroon: A cross-sectional study

**DOI:** 10.1371/journal.pone.0280269

**Published:** 2023-01-20

**Authors:** Frederick Nchang Cho, Yayah Emerencia Ngah, Andrew N. Tassang, Celestina Neh Fru, Peter Canisius Kuku Elad, Patrick Kofon Jokwi, Valmie Ngassam Folefac, Ismaila Esa, Paulette Ngum Fru

**Affiliations:** 1 Cameroon Baptist Convention Health Services – HIV free/Strengthening Public Health Laboratory Systems, Buea, Cameroon; 2 Infectious Disease Laboratory, Faculty of Health Sciences, University of Buea, Buea, Cameroon; 3 District Health Services Bamenda, North West Regional Delegation of Health, Ministry of Health, Buea, Cameroon; 4 Department of Obstetrics and Gynaecology, Faculty of Health Sciences, University of Buea, Buea, Cameroon; 5 Buea Regional Hospital Annex, Buea, Cameroon; 6 Atlantic Medical Foundation, Mutengene, Cameroon; 7 Department of Sociology and Anthropology, Faculty of Social and Management Sciences, University of Buea, Buea, Cameroon; 8 Department of Microbiology and Parasitology, University of Buea, Buea, Cameroon; 9 Department of Biochemistry and Molecular Biology, Faculty of Science, University of Buea, Buea, Cameroon; 10 Sintieh Research Academy, Yaoundé, Cameroon; 11 Department of Public Health and Hygiene, Faculty of Health Sciences, University of Buea, Buea, Cameroon; 12 District Health Services Tiko, South West Regional Delegation of Health, Ministry of Health, Buea, Cameroon; Xiamen University - Malaysia Campus: Xiamen University - Malaysia, MALAYSIA

## Abstract

**Introduction:**

This study aimed to establish pre-/post Coronavirus Disease 2019 (COVID-19) diagnosis/treatment symptoms, ownership/utilisation of face masks (FMs), as well as vaccine hesitancy (VH) amongst patients recovering from COVID-19.

**Methods:**

A cross-sectional survey was conducted from April - October 2021. Data was collected with structured self-administered questionnaires. Multinomial regression was used to determine associations between ownership/utilisation of FMs with respondents’ characteristics.

**Results:**

Unproductive cough and fatigue were prevalent before and after treatment. Pre-/Post COVID-19 symptoms severity ranged from mild to moderate. There was a COVID-19 VH rate of 492 (74%). The prevalence of FM ownership and utilisation were, respectively, 613 (92.2%) and 271 (40.8%). One main factor was associated with FM ownership; respondent’s sex (*p*; 5.5x10^-2^, OR; 0.5, 95%C.I; 0.3 – 1.0). The main reasons for irregular utilisation were; inability to be consistent, only used outdoors, and boredom.

**Conclusion:**

The treatment of COVID-19 does not mean immediate recovery as mild to moderate grade severity still persists. Face mask availability and ownership does not mean appreciable utilisation. This study advocates for an intensification of COVID-19 preventive practices, as well as elaborate education on the importance of vaccination.

## Introduction

A novel enveloped ribonucleic acid (RNA) beta (β) corona virus, Coronavirus Disease 2019 (COVID-19) has spread widely from Wuhan, causing tens of thousands of deaths, especially in patients with severe COVID-19 as from December 2019 [1–5]. The coronavirus diseases vary from mild, self-limiting forms to more severe manifestations depending on the type of viruses involved [6, 7]. As the pandemic progressed, about 461,175,583 cases, 6,071,057 deaths, and 394,441,157 recovered cases have been reported globally [8]. The impacts of the pandemic have been felt unequally around the world, with Europe and America being highly affected, as shown by overwhelmed health systems and high death tolls [9].

The Coronavirus Disease 2019 (COVID-19) is spreading across Africa, and available data indicates that it is on the rise in Cameroon, Uganda, and other African countries [10, 11]. A third wave of the pandemic, characterised by the delta (δ) strain of the virus, has also reached parts of Africa. The first case of COVID-19 in Cameroon was identified on the 6^th^ of March 2020 [12] and as of 6^th^ July 2020, 320 deaths were recorded and almost 15,000 cases were confirmed [13–15]. As of the 29^th^ of December 2021, 3,756 health personnel tested positive for COVID-19 nationwide [16], of whom two of the 45 from the South West Region of Cameroon died [17], and as the situation progressed 121,650 cases, 1,935 dead, and 117,263 recovered nationwide [8, 18]. As of 28^th^ July 2021, 285,522 people had received the first dose of the vaccine and 53,365 the second, representing 38% consumption of the received vaccines [17].

The reference and definitive diagnosis of Severe Acute Respiratory Syndrome Coronavirus-2 (SARS-CoV-2) infection is the reverse transcription polymerase chain reaction assay (rt-PCR). Chest X-ray (CXR), although not generally considered sensitive for the detection of pulmonary abnormalities in the early stage of the disease, can be a useful diagnostic tool for monitoring the rapid progression of lung involvement in COVID-19, especially in patients admitted to intensive care units (ICUs) [19].

Many approaches have been used for the treatment of high mortality risk patients as well as other patients [20, 21]. The recovery of patients presenting with varying symptoms depends on treatment regimens administered [20], as well as the severity of the infection. The clinical spectrum of COVID-19 varies from asymptomatic presentation (Stage I) to moderate to severe states (Stages IIa and IIb) characterized by respiratory failure necessitating, mechanical ventilation and ICU support and those manifesting with critical clinical condition (Stage III), with mild to moderate disease occurs in approximately in 81% of cases [22].

Individuals of all ages are at risk for infection and severe disease. However, the probability of serious COVID-19 disease is higher in the old, those living in nursing homes or long-term care facilities, and those with chronic medical conditions [21].

In Cameroon, SARS-CoV-2 screening, hydroxychloroqine/azithromycin regimens and COVID-19 vaccines are free in all Health Districts while the ADSAK/ELIXIR COVID costs 20,000Fcfa (30.6€ or 36.3$) and is available in Catholic and Private Health Facilities across the Country. As of 8^th^ July 2021, the following COVID-19 vaccines; Sinopharm BIBP, and Oxford–AstraZeneca were in Cameroon, as well as Janssen (Johnson & Johnson) donated by the United States Government, and the African Union [16]. A variety of face masks (FMs) are available in Pro-pharmacies in most health facilities, local shops, tailoring shops, and street vendors. The average cost of FMs was 350Fcfa (0.53€ or 0.63$); range 200 – 500Fcfa (0.31 – 0.76€ or 0.36 – 0.91$). The majority of the population using FMs, use the locally made masks/cloth masks that costs 200Fcfa (0.31€ or 0.31$). The monthly income of the population ranges between 16.65 and 72.11 € (or 19.81 and 85.81$) [23]. Conversion rates; 1€ = 655Fcfa, 1$ = 551.13Fcfa.

Cameroon is known as Africa in miniature, with variety of ethnic groups. Apart from the conflict hit areas, Cameroon is an economic hub in Central Africa with an estimated population of about 23,344,000 in 2015 [23]. Due to the pandemic, a couple of studies on COVID-19 have been carried out in Cameroon; vaccine hesitancy/acceptance [24–26], responses to COVID-19 in the educational sector [13], **clinical characteristics and outcomes of patients hospitalised for COVID-19** [27, 28], COVID-19 preventive behaviours [12], as well as knowledge attitudes and practices [29, 30], but none has explored the diagnosis/treatment symptoms, face mask ownership/utilisation, preventive measures, and vaccine hesitancy amongst patients recovering from the COVID-19 infection. Thus, in this study, we aimed to establish pre- and post-COVID-19 diagnosis/treatment symptoms amongst persons who suffered from COVID-19 infection, ownership and utilisation of face masks, as well as vaccine hesitancy.

## Methods

### Study design and setting

A cross-sectional study was conducted from April 29^th^ - July 4^th^, and August 2^nd^ - October 30^th^, 2021 amongst persons who had tested positive for COVID-19. Patients who admitted having recovered from COVID-19 as confirmed by a negative control test result, were enrolled into this study. Those who consented to fill the questionnaire, were cautioned not to fill it if they had earlier filled a similar questionnaire, online. Information on pre-/post-recovery clinical symptoms were collected with the use of anonymous online as well as ‘hand-filled’ questionnaires (S1 Appendix).

Bafoussam, Douala, and Yaoundé are metropolis located respectively in the West, Littoral, and Centre regions of Cameroon, with Douala, and Yaoundé being the largest.

### Study population and target sample size

The study population was persons who by chance had new technology devices and could access social media platforms, and those who were at home/work place/outpatient department (OPD) of selected hospitals. The Google© questionnaire’s link was distributed via social media: WhatsApp, LinkedIn, Facebook, Skype, and Instagram. It was also shared from door-to-door, work places and OPD of hospitals in the towns of Bafoussam, Douala, and Yaoundé. Being COVID-19 positive, adult (≥ 20 years of age) and verbal consent to participate in the study constituted the inclusion criteria.

An estimated minimum sample size of 256 per town was calculated with the CDC Epi Info 7 StatCalc, and collected by convenience sampling, considering the following characteristics: an estimated Cameroon population size of 23,344,000 in 2015 [23], an assumed frequency of persons who have recovered from COVID-19 of 50%, accepted error margin of 5%, and design effect of 2.0.

### Sampling method

The study was conducted during the second wave of the COVID-19 pandemic in Cameroon. The convenience sampling technique was implored wherein participants were approached and informed of the study objectives via social media platforms, at their work places, at their door steps and OPD of hospitals. Investigators continuously posted the questionnaire together with reminders on various platforms, regularly checked incoming responses, as well as regularly checked workplaces, homes, and OPDs to collect ‘hand dropped’ questionnaires.

### Data collection and analysis

Data was collected with the use of anonymous self-administered online [3, 4] and workplace/door-to-door/OPD questionnaires using Microsoft Office Excel and analysed with CDC Epi Info version 7.2. The questionnaire that consisted of 29 questions, aimed to collect information on: socio-demographic characteristics, ownership/utilisation of FMs, pre-/post-symptoms, and treatment of COVID-19, as well as opinions on COVID-19 vaccination. The survey instrument took approximately 10 minutes to complete. The validity of the questionnaire was confirmed by pre-testing in 10 participants who were excluded from the study. Based on the pre-test study, the format and wording of some questions were corrected and refined. Data from the 10 participants was used to assess internal consistency reliability using Cronbach’s alpha (α) [31–33]. The results showed adequate internal consistency reliability (with Cronbach’s α = 0.72) [31, 32].

Age groups, sex, education, and occupation were summarised as counts and percentages. Body Mass Index (BMI), age, household size, number of sleeping rooms, length of hospital stay/quarantine, were expressed as ranges and means. Analysis of variance (ANOVA) and Multinomial Logistic Regression were used to determine associations between hospitalisation/quarantination and ownership/utilisation of FMs with demographic characteristics. In order to control for confounders, the least significant independent covariates were excluded from the Multinomial Logistic Regression analysis. The following models were used:

Ownership of FM = β_0_ + β_1_Age + β_2_Sex + β_3_Marital Status + β_4_Education + β_5_Occupation + β_6_Family size + ε, and

Household ownership of FM/Correct wearing of FM/ = β_0_ + β_1_Age + β_2_Sex + β_3_Marital Status + β_4_Education + β_5_Occupation + β_6_Family size + β_7_Residential area + ε. Where β_0_ is a constant, β_1_, β_2_, β_3_, …, and β_7_ are coefficients and ε is the regression error.

The significance level was set at *<* 0.05.

### Definition of concepts/dependent variables

#### Severity of COVID-19

The severity grading of COVID-19 in this study consisted of pre-/post-treatment symptoms and their duration, age (≥60 years considered riskier), and comorbidities (five; on a score of 2) with a severity grading of < 20% for asymptomatic, 20 - < 40% for mild, 40 - < 80% for moderate and ≥ 80% for severe COVID-19.

#### Ownership of face masks

The proportion of respondents who had at least one FM, where the numerator comprised the number of respondents surveyed with at least one FM and the denominator, the total number of respondents surveyed. *Entire household ownership of FMs*; the proportion of households wherein all *de facto* members have FMs, where the numerator comprises the number of households wherein all *de facto* members own FMs and the denominator, the total number of respondents surveyed.

#### Utilisation of face masks

*The correct wearing of FMs*; the proportion of respondents who correctly wore FM, where the numerator comprises the number of respondents surveyed correctly wearing FMs as recommended by the WHO [34] and the denominator, the total number of respondents surveyed. *Adequate utilisation of FMs*; the proportion of respondents who wore at least six or more (≥6) FMs per week, where the numerator comprises the number of respondents surveyed adequately wearing FMs and the denominator, the total number of respondents surveyed. *Regular utilisation of FMs*; the proportion of respondents who regularly wore their FMs, where the numerator comprises the number of respondents surveyed regularly wearing FMs and the denominator, the total number of respondents surveyed.

#### Knowledge related to COVID-19 symptoms

Knowledge related to symptoms of COVID-19 comprised one open-ended question with the possibility of listing 10 or more options. The options were developed by considering previous studies with a similar research theme [35]. The options were sorted in the form of yes or no; if the answer was yes/no, a score of ‘1’/‘0’ was accorded to the participant. Modified Bloom’s cut-off points were used to judge knowledge as very poor (< 20%), poor (20 - < 40%), moderate (40 - < 60%), good (60 - < 80%), and very good (≥ 80%) [7].

#### Practice regarding COVID-19 prevention

Prevention practices consisted of the correct and adequate wearing of FMs, and a question on preventive measures towards COVID-19 (one correct point listed by the respondent earned a score of 1 and an omission or wrong point listed earned a score of 0). Modified Bloom’s cut-off points were used to judge knowledge as very poor (< 20%), poor (20 - < 40%), moderate (40 - < 60%), good (60 - < 80%), and very good (≥ 80%) [7].

#### COVID-19 Vaccine hesitancy

The proportion of respondents who will not voluntarily take COVID-19 vaccine, where the numerator comprises the number of respondents who will not take the vaccine [36] and the denominator, the total number of respondents surveyed.

### Ethical consideration

This study was conducted in accordance with the Helsinki Declaration [37] as well as the principles of Personal Information Protection and Electronic Documents Act [38], and cleared by the North West Regional Delegation of Public Health (Reference N^o^: 95/ATT/NWR//RDPH/BRIGAD). Only respondents who verbally consented to the study, were allowed to participate by filling and submitting the anonymous questionnaire.

## Results

### Characteristics of study population

Six hundred and ninety (690) persons were logged into our Microsoft Excel sheet, from whom 25 were dropped as outliers for age, BMI, and prolonged hospitalisation. Of the 665 persons included in this study, 2,126 household residents were counted; 1,751 (82.4%) were persons 0 – 59 years old and 375 (17.6%) were persons ≥ 60 years old. Four hundred and thirty-seven (52.2%) persons were females and 318 (47.8%) were males (Table 1).

**Table 1:** Characteristics of study participants.

General characteristic	Subclass	Count (%)
**Age groups (in years)**	≤ 40	510 (76.7)
	> 40	155 (23.3)
	Mean age ($\bar{x}$ ± SD)	34.00 ± 9.00
**Sex**	Female	437 (52.2)
	Male	318 (47.8)
**BMI (Kg/m** ^**2**^ **)**	Eutrophic	256 (38.5)
	Overweight	233 (35.0)
	Obese	176 (26.5)
	Mean BMI ($\bar{x}$ ± SD)	27.00 ± 4.00
**Marital status**	Not married	390 (58.6)
	Married	275 (41.4)
**Education**	Primary	142 (21.4)
	Secondary	218 (32.8)
	Tertiary	305 (45.9)
**Occupation**	Business/Private sector	118 (17.7)
	Student	97 (14.6)
	Unemployed	118 (17.7)
	Unskilled worker	64 (9.6)
	Skilled worker	268 (40.3)
**Household size**	1 – 4	546 (82.1)
	5 – 9	119 (17.9)
	Mean household size ($\bar{x}$ ± SD)	3.00 ± 1.00
**Number of sleeping rooms**	One	209 (31.4)
	Two	365 (54.9)
	More than two (3 - 5)	91 (13.7)
	Mean number of rooms ($\bar{x}$ ± SD)	1.00 ± 0.00
**Comorbid condition**	Gastritis	202 (30.4)
	Previously operated	89 (13.4)
	High blood pressure	108 (16.2)
	Hepatitis/Liver disease	95 (14.3)
	Diabetes	50 (7.5)
**Number of comorbidities**	None	202 (30.4)
	At least one	384 (57.7)
	At most two	77 (11.7)

BMI; Body Mass Index [Eutrophic (18.5 ≤ BMI ≥ 24.9), Overweight (25.0 ≤ BMI ≥ 29.9), Obese (BMI ≥ 30.0)], SD; Standard Deviation.

The mean age of the participants was 34 years (SD 9.0, range 20 – 64). Three hundred and ninety (58.6%) were singles, 308 (45.9%) were of tertiary educational status, and 268 (40.3%) were skilled workers.

### Clinical profile of respondents

#### Pre- and post- COVID-19 diagnosis/treatment symptoms

**Fig 1:**
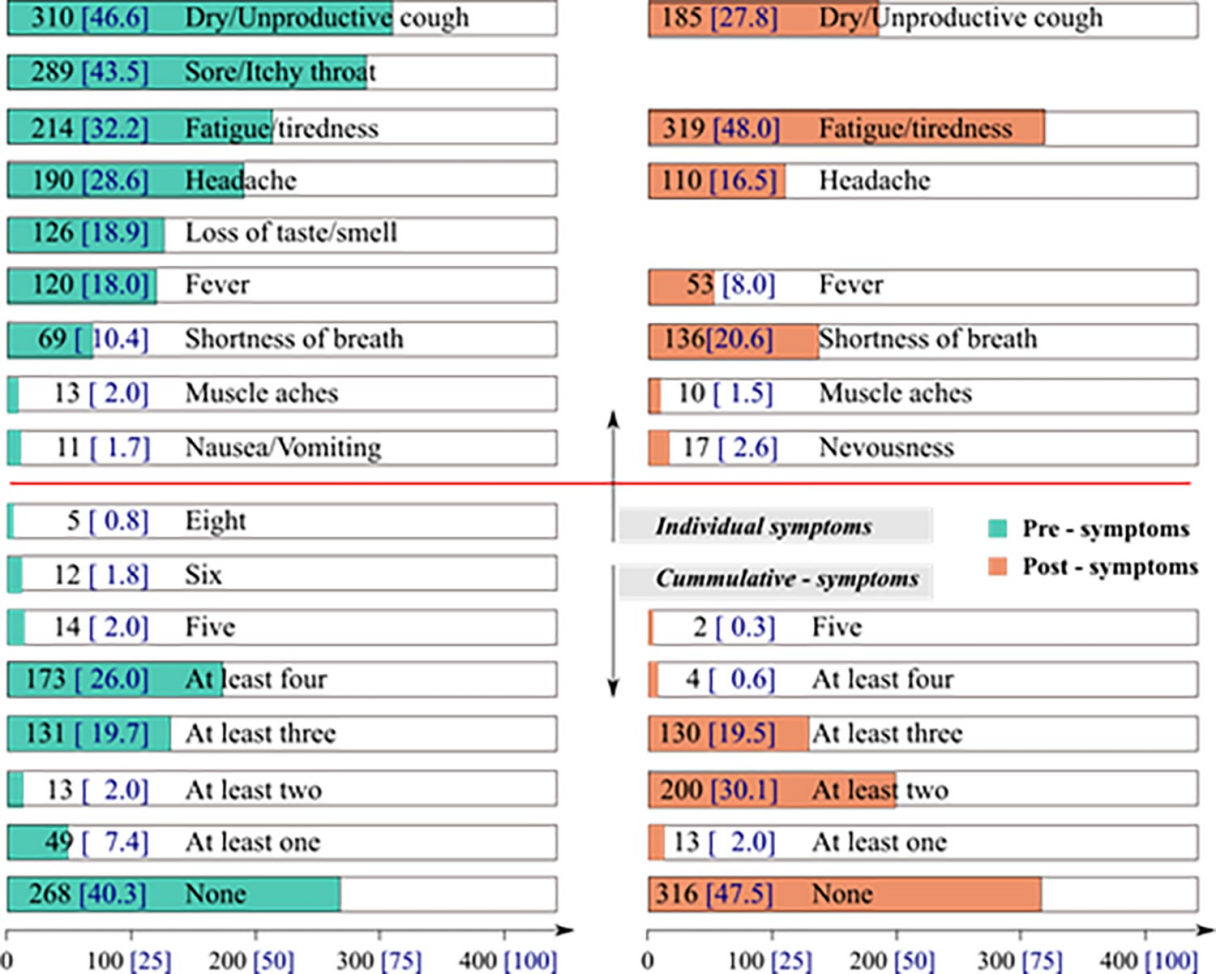
Pre- and post-symptoms of COVID-19.

Three hundred and ten (46.6%), 289 (43.5%), and 214 (32.2%) of the respondents presented with cough, sore throat, and tiredness, respectively (Fig 1) for a mean duration of 2.30 days (SD 2.46, range 0 – 9 days) prior to diagnosis and treatment. Fatigue/Tiredness, dry/unproductive cough, and shortness of breath were experienced for a mean duration of 3.65 days (SD 3.17, range 0 – 8) after drug administration.

Two hundred and sixty-eight (40.3%) and 316 (47.5%) of the respondents, respectively, manifested with no symptoms of COVID-19 prior to testing positive and after treatment (Fig 1).

#### Severity and comorbidities

**Fig 2:**
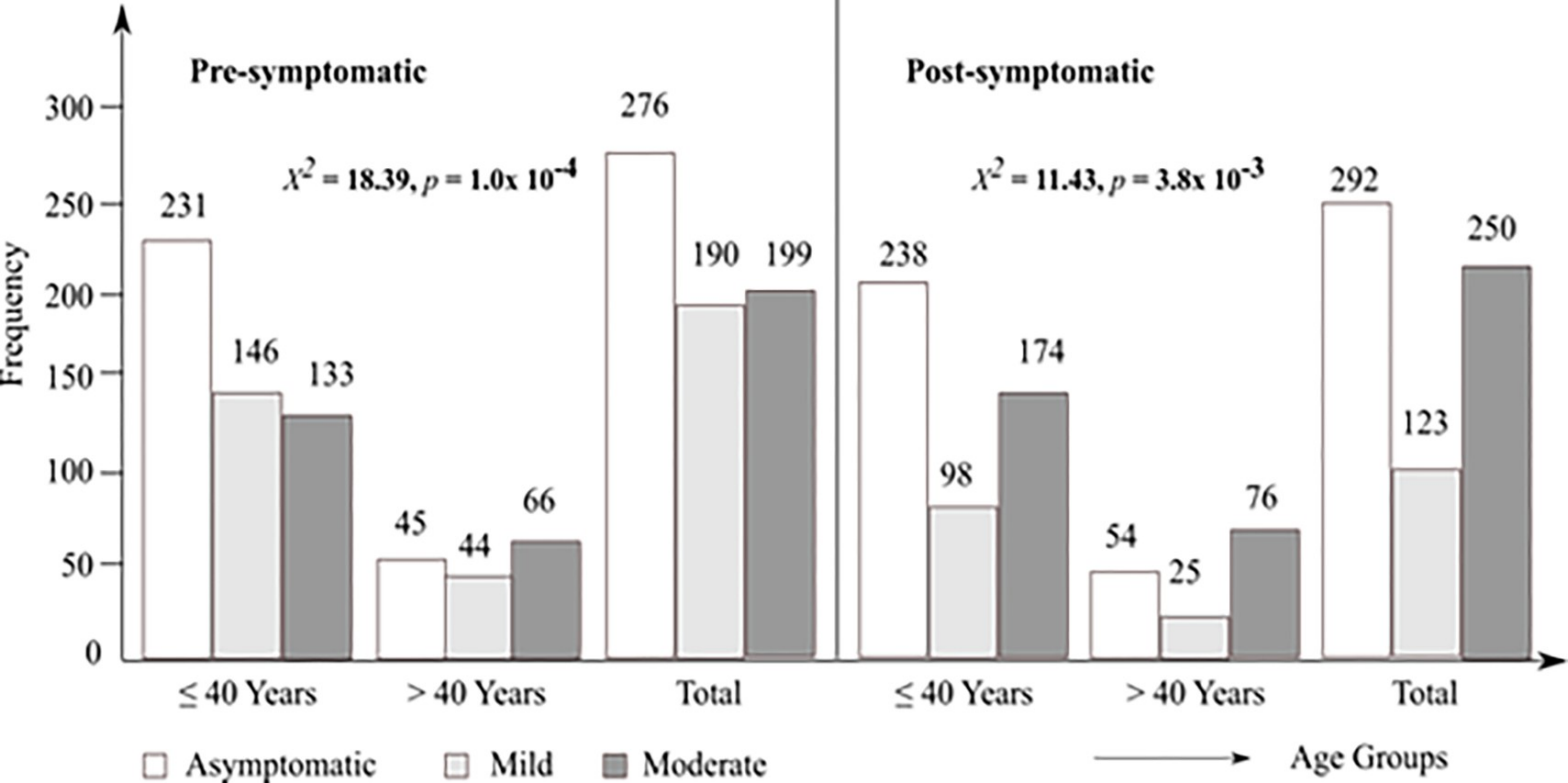
Severity grading for pre- and post-COVID-19 amongst age groups.

The severity of pre- and post-COVID-19 diagnosis and treatment are presented in Fig 2. The most common comorbidities were gastritis 202 (30.4%), high blood pressure 108 (16.2%), and hepatitis and/or other liver diseases 95 (14.3%), with 202 (30.4%) having no comorbidity and 77 (11.7%) having at most two comorbidities (Table 1).

#### Duration of hospitalisation/quarantine

The mean duration of hospitalisation and quarantine periods were, respectively, 2.95 days (SD 7.20, range 0 – 24) and 6.84 days (SD 7.00, range 0 – 28). Male respondents stayed longer in the hospital and in quarantine than females (Table 2).

**Table 2:** Association of hospitalisation/quarantination with respondents’ characteristics; ANOVA.

		Hospitalisation (*in days*)		Quarantination (*in days*)	
**Variable**	**Subclass**	$\bar{x}$ ± SD	F (*p* – value)	$\bar{x}$ ± SD	F (*p* – value)
**Age (in years)**	≤ 40	2.92 ± 7.20	2.0x10^-2^ (8.6x10^-1^)	6.70 ± 7.03	1.1x10° (3.0x10^-1^)
	> 40	3.02 ± 7.12		7.35 ± 6.74	
**Sex**	Female	2.62 ± 6.84	1.5x10^-1^ (2.2x10^-1^)	6.81 ± 6.93	1.2x10^-2^ (9.1x10^-1^)
	Male	3.30 ± 7.48		6.87 ± 7.02	
**BMI (kg/m^2^)**	Eutrophic	2.52 ± 6.79	2.8 (2.4x10^-1^)	6.58 ± 6.65	3.0x10^-1^ (**5.3x10^-2^**)
	Overweight	3.30 ± 7.56		7.05 ± 6.71	
	Obese	3.13 ± 7.14		6.95 ± 7.74	
	Total	2.95 ± 7.20		6.84 ± 7.00	

**Bold** numbers are significant *p* – values. BMI; Body Mass Index [Eutrophic (18.5 ≤ BMI ≥ 24.9), Overweight (25.0 ≤ BMI ≥ 29.9), Obese (BMI ≥ 30.0)]

#### Care, treatment and vaccination hesitancy

Patients received either the Ministry of Health’s (MOH) protocol of oral hydroxychloroquine, paracetamol, vitamin C, zinc, and azithromycin or the local remedy ADSAK/ELEXIR COVID or both. Depending on the severity and comorbidities, some patients received or added extra doses to the prescribed doses.

Five hundred and seventy-three (86.2%) of the respondents were prescribed and administered the hydroxychloroqine/Azithromycin/Paracetamol/Zinc/Vitamin C regimen, 25 (3.8%) were simply advised to go home, and 230 (34.6%) opted to supplement the MOH’s regimen with the local remedy ADSAK/ELIXIR COVID (Table 3).

**Table 3:** Treatment regimens for respondents.

Treatment	Frequency	Percent
Counselled to go home for self-quarantine	25	3.8
Hydroxychloroqine/Azithromycin/Paracetamol/Zinc/Vitamin C	573	86.2
ADSAK/ELIXIR COVID	230	34.6
**Cumulative treatment**		
None	81	12.2
At least one treatment regimen	340	51.1
Both treatment regimens	244	36.7
Supplemented medication	260	39.1

**Fig 3:**
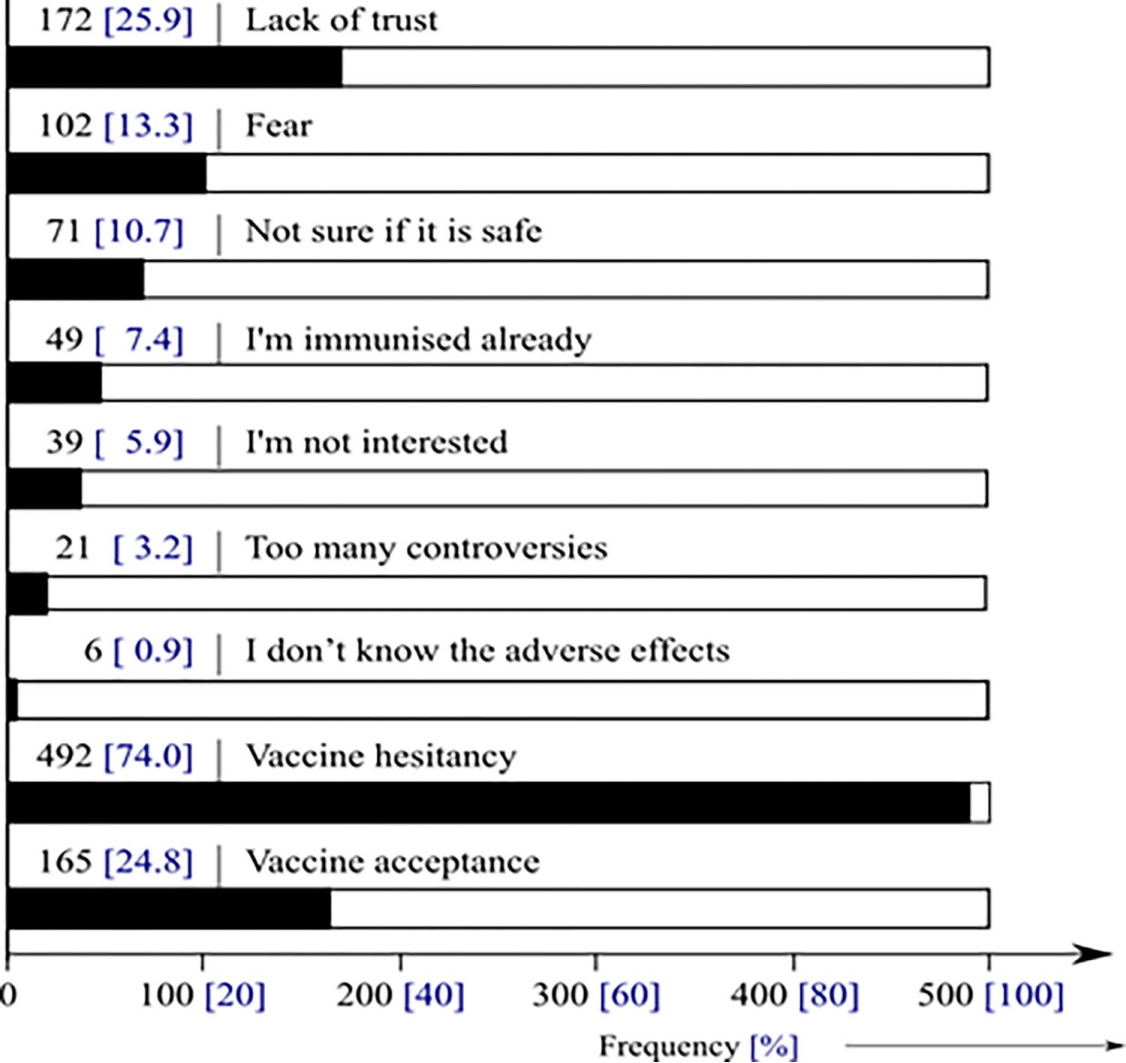
COVID-19 Vaccine hesitancy and reasons for hesitancy.

Only 165 (24.8%) of the respondents admitted that they will accept the vaccine when it is feasible, thus yielding a COVID-19 hesitancy rate of 74% (Fig 3). From multinomial regression analysis, the odds for denying COVID-19 vaccine was higher amongst the unemployed (OR; 1.5, 95%C.I; 0.8 – 3.0), unskilled workers (OR; 1.4, 95%C.I; 0.7 – 3.0), and those residing in Bamenda and Bafoussam [(OR; 1.3, 95%C.I; 0.6 – 2.5) vs (OR; 1.2, 95%C.I; 0.6 – 2.4)], when compared with their counterparts (S3 Table in S1 File).

### Ownership/Utilisation and sources of face masks

#### Ownership/Utilisation of face masks

**Fig 4:**
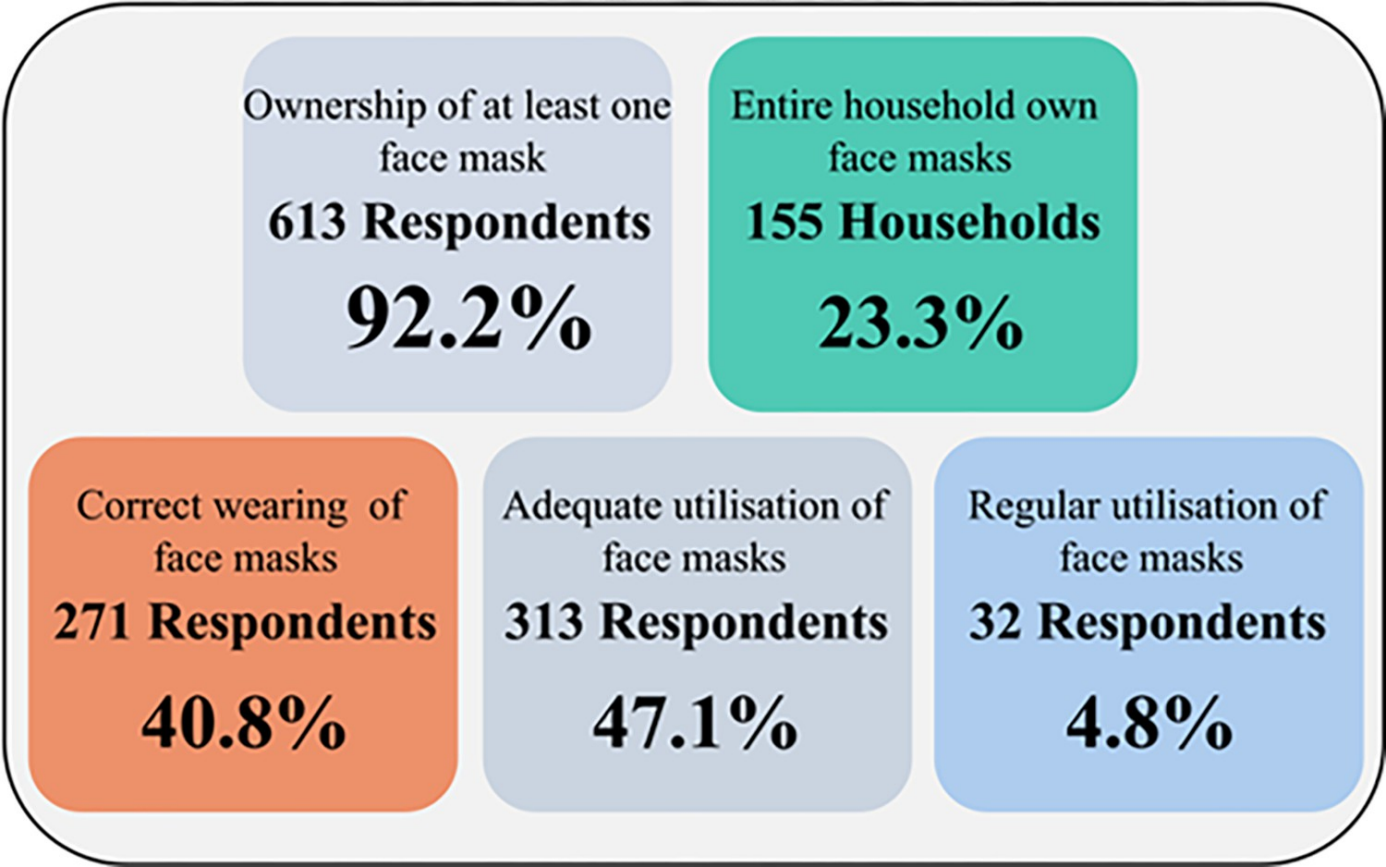
Ownership/utilisation of face masks.

Of the 665 respondents sampled, 155 (23.3%) were in households wherein all residents owned FMs (Fig 4).

Two hundred and seventy-one (40.8%) of the respondents correctly wore their FMs to cover the nose, mouth, and chin, such so as to be able to breathe, while 313 (47.1%) adequately/effectively used FMs for ≥ 6 days/week (Fig 4).

#### Factors associated with ownership/utilisation of face masks

Multinomial logistic regression was used to test if respondent characteristics were significantly associated with face mask ownership/utilisation. The results indicated that the odds for the ownership of FMs by the entire household were higher amongst the male sex (OR; 1.1, 95%C.I; 0.7 – 1.5), married persons (OR; 1.3, 95%C.I; 0.8 – 2.0), primary school leavers (OR; 1.3, 95%C.I; 0.6 – 2.6), students as well as business operators [(OR; 1.6, 95%C.I; 0.9 – 3.1) vs (OR; 1.3, 95%C.I; 0.7 – 2.1)] and Bamenda as well as Buea [(*p*; <1.0x10^-3^, OR; 7.6, 95%C.I; 3.8 – 15.5) vs (*p*; <1.0x10^-3^, OR; 14.6, 95%C.I; 6.5 – 33.1)] (Table 4).

**Table 4:** Association of respondents’ characteristics with face mask ownership/utilisation.

	Ownership of FM (*n* = 613)			Entire household own FM (*n* = 155)		
**Characteristic**	***n* (%)**	***p*-value**	**OR (95% C.I)**	***n* (%)**	***p*-value**	**OR (95% C.I)**
**Age groups (in years)**						
≤ 40/> 40	472/141	2.6x10^-1^	1.5 (0.7 – 3.1)	110/45	8.7x10^-1^	1.0 (0.6 – 1.8)
**Sex**						
Male/Female	286/327	**4.5x10^-2^**	0.5 (0.3 – 1.0)	76/79	9.1x10^-1^	1.1 (0.7 – 1.5)
**Marital status**						
Married/Not married	253/360	2.9x10^-1^	0.7 (0.4 – 1.3)	68/87	2.4x10^-1^	1.3 (0.8 – 2.0)
**Education**						
Secondary/Tertiary	198/283	4.1x10^-1^	0.7 (0.4 – 1.5)	42/89	9.6x10^-1^	1.0 (0.5 – 1.6)
Primary/Tertiary	132 (21.5)	9.7x10^-1^	1.1 (0.4 – 3.0)	24 (15.5)	5.6x10^-1^	1.3 (0.6 – 2.6)
**Occupation**						
Student/Skilled worker	87/244	6.2x10^-1^	0.8 (0.3 – 1.9)	27/69	1.3x10^-1^	1.6 (0.9 – 3.1)
Unemployed/Skilled worker	108 (17.6)	7.0x10^-1^	0.8 (0.3 – 2.3)	16 (10.3)	4.3x10^-1^	0.7 (0.3 – 1.6)
Unskilled/Skilled worker	61 (9.9)	4.9x10^-1^	1.6 (0.4 – 6.5)	6 (3.9)	1.8x10^-1^	0.5 (0.2 – 1.4)
Business operator/Skilled worker	113 (18.4)	2.1x10^-1^	2.0 (0.7 – 4.4)	37 (23.9)	3.9x10^-1^	1.3 (0.7 – 2.9)
**Household size**						
5 – 9/1 – 4	115/498	**4.1x10^-2^**	**3.2** (1.1 – 9.6)	27/128	7.0x10^-2^	0.6 (0.3 – 1.0)
**Residence**						
Bamenda/Yaoundé	-	**-**	**-**	31/44	**<1.0x10^-3^**	**7.6** (3.8 – 15.5)
Bafoussam/Yaoundé	-	**-**	**-**	8 (5.2)	6.3x10^-1^	0.8 (0.3 – 1.9)
Buea/Yaoundé	-	**-**	**-**	35 (22.6)	**<1.0x10^-3^**	**14.6** (6.5 – 33.1)
Douala/Yaoundé	-	**-**	**-**	37 (23.9)	2.4x10^-1^	0.7 (0.5 – 1.2)
	**Correct wearing of FM (*n* = 271)**			**Adequate utilisation of FM (n 313**		
**Age groups (in years)**						
≤ 40/> 40	209/62	9.9x10^-1^	1.0 (0.7 – 1.5)	250/63	**2.2x10^-3^**	1.9 (1.3 – 2.9)
**Sex**						
Male/Female	119/152	1.2x10^-1^	0.8 (0.6 – 1.1)	152/161	7.1x10^-1^	**1.1** (0.8 – 1.4)
**Marital status**						
Married/Not married	103/168	**1.3x10^-2^**	0.6 (0.5 – 1.0)	123/190	6.1x10^-2^	0.7 (0.5 – 1.0)
**Education**						
Secondary/Tertiary	74/133	**4.1x10^-2^**	0.6 (0.4 – 1.0)	99/152	7.9x10^-1^	0.9 (0.6 – 1.4)
Primary/Tertiary	64 (23.6)	9.5x10^-1^	1.1 (0.6 – 1.8)	62 (19.8)	7.4x10^-1^	0.9 (0.5 – 1.6)
**Occupation**						
Student/Skilled worker	35/114	5.5x10^-1^	0.8 (0.5 – 1.4)	39/149	**5.0x10^-4^**	0.4 (0.2 – 0.6)
Unemployed/Skilled worker	55 (20.3)	6.1x10^-1^	1.2 (0.6 – 2.1)	55 (17.6)	8.2x10^-2^	0.5 (0.3 – 1.1)
Unskilled/Skilled worker	25 (9.2)	9.4x10^-1^	1.0 (0.5 – 1.9)	29 (9.3)	8.6x10^-2^	0.5 (0.3 – 1.1)
Business operator/Skilled worker	42 (15.5)	2.9x10^-1^	0.8 (0.5 – 1.2)	41 (13.1)	1.0x10^-4^	0.3 (0.2 – 0.6)
**Household size**						
5 – 9/1 – 4	49/222	6.2x10^-1^	1.1 (0.7 – 1.7)	60/253	8.7x10^-2^	1.4 (0.9 – 2.3)
**Residence**						
Bamenda/Yaoundé	15/95	2.4x10^-1^	0.6 (0.3 – 1.3)	19/121	5.9x10^-2^	0.5 (0.3 – 1.0)
Bafoussam/Yaoundé	30 (1.1)	**1.1x10^-2^**	2.3 (1.2 – 4.6)	26 (8.3)	7.1x10^-1^	0.9 (0.4 – 1.7)
Buea/Yaoundé	19 (7.0)	9.3x10^-1^	1.0 (0.5 – 1.9)	23 (7.4)	9.7x10^-1^	1.0 (0.5 – 2.0)
Douala/Yaoundé	112 (41.3)	6.1x10^-1^	1.1 (0.8 – 1.6)	124 (39.6)	4.0x10^-1^	0.8 (0.6 – 1.2)

OR = Odds Ratio; C.I. = Confidence Interval; **Boldface** numbers indicate significant *p* values.

Respondents with the primary level of education (*p*; 9.5x10^-1^, OR; 1.1, 95% C.I; 0.6 – 1.8), were more likely to correctly use FMs than those with the tertiary educational status. More females had FMs than males and were less likely to correctly wear them compared to males (*p*; 1.2x10^-1^, OR; 0.8, 95% C.I; 0.6 – 1.1) (Table 4).

**Fig 5:**
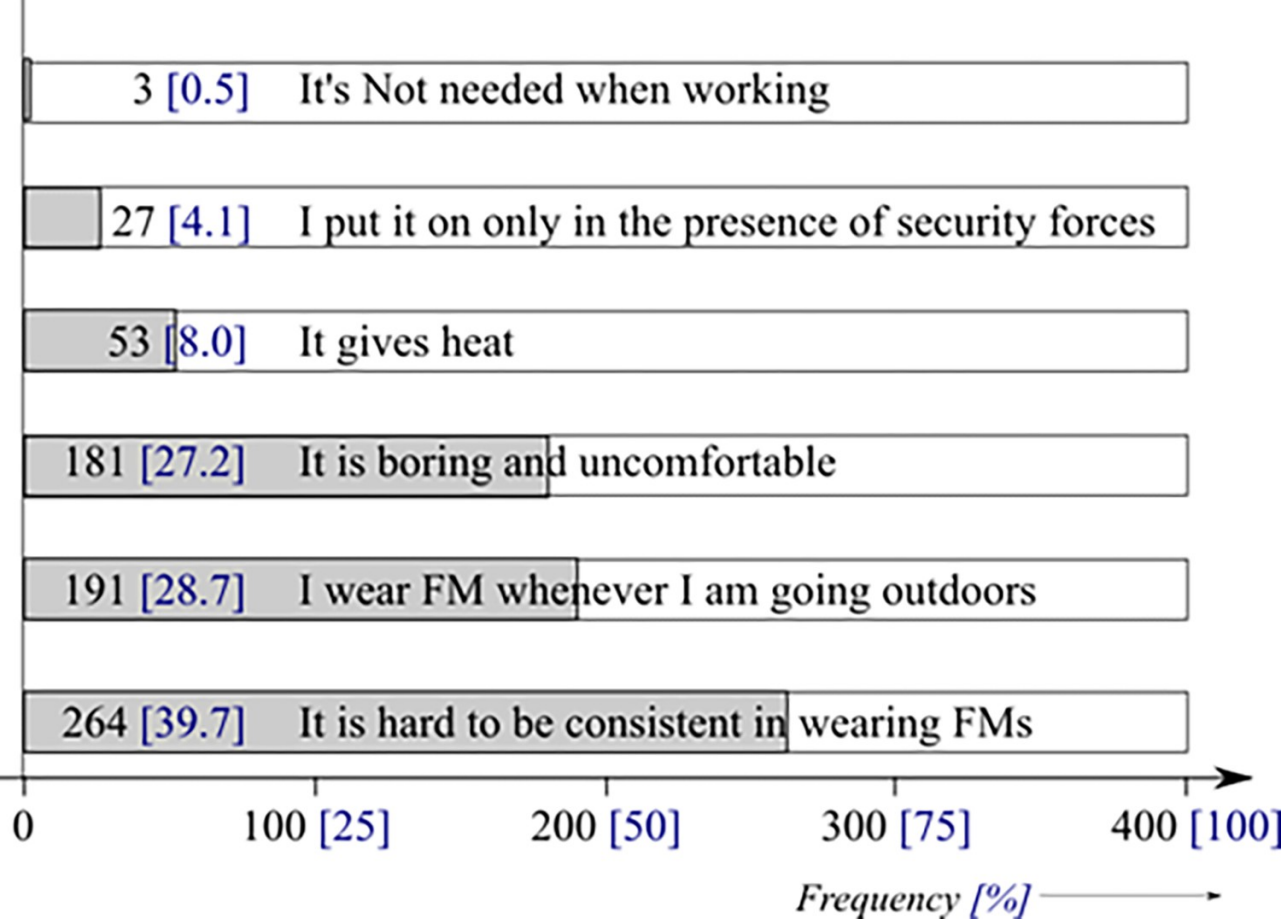
Reasons for irregular use of face masks.

The reasons advanced for the irregular use of FMs from respondents’ perspective were: ‘it is hard to be consistent’ (39.7%), ‘I wear them whenever I am going outdoors’ (28.7%), and ‘it is boring/uncomfortable’ (27.2%) (Fig 5).

#### Source of face masks

Respondents either purchased FMs, 58.8% or obtained them freely from the office/workplace 35.8%, and a gift from a relationship 35.2%, or self-made 17.3% (Table 5).

**Table 5:** Sources of face masks.

Source of face masks	Frequency	Percent
**A gift from a relationship**	234	35.2
**Purchased from the shop/Pharmacy**	238	35.8
**From the office/Workplace**	391	58.8
**Self-made/Tailor**	115	17.3

### Knowledge of COVID-19 symptoms and household preventive measures against it

#### Knowledge of COVID-19 symptoms

Of the 10 symptoms anticipated, 21 (3.2%) of the respondents indicated ignorance, while 258 (38.8%) were informed on all 10 (S1 Table in S1 File). Four hundred and forty (66.2%) of the respondents have very good knowledge of COVID-19 symptoms, with a knowledge score of ≥80%. Participants, 40 years or less (*p*; 1.7x10^-2^, OR; 1.7, 95% C.I; 1.1 - 2.6), unskilled workers as well as business operators [(OR; 1.3, 95% C.I; 0.6 - 2.7) vs (OR; 1.2, 95% C.I; 0.7 - 2.0)], and those residing in Buea as well as Douala [(OR; 1.3, 95% C.I; 0.6 - 2.9) vs (OR; 1.1, 95% C.I; 0.7 - 1.6)], higher odds to have very good knowledge of COVID-19 symptoms when compared with their counterparts (Table 6).

**Table 6:** Association of respondent’s characteristics with knowledge of COVID-19 symptoms/household preventive measures of COVID-19.

Respondent’s Characteristic	VG Knowledge of symptoms (n = 440)			Moderate HH preventive measures (n = 222)		
**Age groups (in years)**	***n* (%)**	***p*-value**	**OR (95% C.I)**	***n* (%)**	***p*-value**	**OR (95% C.I)**
≤ 40/> 40	344/96	**1.7x10^-2^**	1.7 (1.1 - 2.6)	175/47	1.2x10^-1^	1.4 (0.9 - 2.2)
**Sex**						
Male/Female	200/240	3.6x10^-1^	0.8 (0.6 - 1.2)	96/126	1.9x10^-1^	0.8 (0.6 - 1.1)
**Marital status**						
Married/Not married	185/255	8.0x10^-1^	0.9 (0.6 - 1.4)	89/133	2.7x10^-1^	0.8 (0.6 - 1.1)
**Education**						
Secondary/Tertiary	142/205	5.0x10^-1^	0.9 (0.6 - 1.3)	72/104	5.8x10^-1^	0.8 (0.6 - 1.3)
Primary/Tertiary	93 (21.1)	1.9x10^-1^	0.6 (0.4 - 1.2)	46 (20.7)	4.9x10^-1^	0.8 (0.4 - 1.5)
**Occupation**						
Student/Skilled worker	53/182	**3.8x10^-3^**	0.5 (0.3 - 0.8)	24/96	**8.0x10^-3^**	0.5 (0.3 - 0.8)
Unemployed/Skilled worker	75 (17.1)	6.0x10^-1^	0.8 (0.5 - 1.6)	36 (16.2)	2.0x10^-1^	0.7 (0.4 - 1.2)
Unskilled/Skilled worker	47 (10.7)	4.8x10^-1^	1.3 (0.6 - 2.7)	24 (10.8)	8.4x10^-1^	0.9 (0.5 - 1.8)
Business operator/Skilled worker	83 (18.9)	5.1x10^-1^	1.2 (0.7 - 2.0)	42 (18.9)	8.5x10^-1^	0.9 (0.6 - 1.5)
**Household size**						
5 – 9/1 – 4	76/364	6.0x10^-1^	0.9 (0.6 - 1.4)	40/182	5.1x10^-1^	1.7 (0.7 - 1.8)
**Residence**						
Bamenda/Yaoundé	21/167	**4.0x10^-4^**	0.3 (0.2 - 0.6)	9/87	**1.0x10^-2^**	0.4 (0.2 - 0.8)
Bafoussam/Yaoundé	31 (7.0)	**4.5x10^-2^**	0.5 (0.3 - 1.0)	14 (6.3)	7.8x10^-2^	0.5 (0.3 - 1.1)
Buea/Yaoundé	35 (7.9)	4.6x10^-1^	1.3 (0.6 - 2.9)	13 (5.9)	1.7x10^-1^	0.6 (0.3 - 1.2)
Douala/Yaoundé	186 (42.3)	6.8x10^-1^	1.1 (0.7 - 1.6)	99 (44.6)	7.4x10^-1^	1.1 (0.7 - 1.6)

OR = Odds Ratio; C.I. = Confidence Interval; VG = Very Good; HH = Household; **Boldface** numbers indicate significant *p* values.

#### Household preventive measures of COVID-19

Of the five preventive measures, 570 (85.7%) of the respondents pointed out to regular hand washing, 75 (11.3%) made no mention of any of the measures, while 110 (16.5%) made mention of four of the measures (S2 Table in S1 File).

Two hundred and twenty-two (8.4%) implemented moderate (≥50%) preventive measures against COVID-19. The odds of implementing COVID-19 preventive measures was higher amongst those who were 40 years or less (OR; 1.4, 95%C.I; 0.9 - 2.2), students (*p*; 8x10^-3^, OR; 0.5, 95%C.I; 0.3 - 0.8), and those residing in Bamenda (*p*; 1x10^-2^, OR; 0.4, 95%C.I; 0.2 - 0.8), when compare with those older than 40 years, skilled workers, and those residing in Yaoundé respectively (Table 6).

## Discussion

Our study adds to the description of COVID-19 in Cameroon where there is a large gap in the literature. Our study revealed that; pre-/post symptoms were dry cough and tiredness, with sore throat and headache occurring more before diagnosis/treatment while shortness of breath persisted after treatment. Gastritis, high blood pressure, and hepatitis were the underlying comorbidities of COVID-19 amongst respondents. Majority of the respondents owned FMs, few households owned FMs, while the use of FMs was generally low in all aspects. Many respondents feared COVID-19 vaccines leading to a very high VH rate.

### Pre- and post – COVID-19 diagnosis/treatment symptoms

Cough, tiredness, and headache occurred in patients before and after treatment. A study from Ethiopia proved that these symptoms were confirmed amongst COVID-19 cases, with other common symptoms being sore throat [27]. Fever, fatigue, and cough, however, remain the most common symptoms in many cohort studies [28, 39, 40].

Disease severity ranged from asymptomatic (41.5% vs 43.9%) to mild (28.6% vs 18.5%) to moderate (29.9% vs 37.6%) before and after treatment. This was similar to findings reported in Ethiopia [27] but different from the findings of another study in Cameroon [28]. Nearly two-thirds of the patients suffered from at least one comorbidity (57.7%), of which gastritis was the most common (30.4%). This was similar to the 29% reported in Ethiopia [27], and different from the findings of another study [41], in which it was asserted that the most common comorbidities were obesity, diabetes and hypertension. These differences may be as a result of differences in study population and study design.

The mean duration of hospitalisation and quarantine period were, respectively, 2.95 days (SD 7.20, range 0 – 24) and 6.84 days (SD 7.00, range 0 – 28). This was lower compared with mean hospitalisation of five days reported in Cameroon [28] and the 13.5 – 42 days (SD, 9.7) reported in studies elsewhere [27, 39, 40]. Recovery, however, depends on the severity of the infection as well as comorbidities. Severity seems to have dropped with time, thus leading to both little hospitalisation and quarantine periods.

Five hundred and seventy-three (86.2%) of the respondents were prescribed and administered the hydroxychloroqine/Azithromycin/Paracetamol/Zinc/Vitamin C regimen, 3.8% were counselled to go home. In a similar study, all patients received a treatment protocol with oral chloroquine, paracetamol, vitamin C, zinc, amoxicillin combined with clavulanic acid, and azithromycin. Further, some patients received anticoagulants, corticosteroids, or intravenous antibiotics, with 15% of confirmed cases undergoing non-invasive ventilation [27].

### COVID-19 vaccine hesitancy/acceptance

In this study, vaccine hesitancy (VH) was 74.0% (vaccine acceptance of 24.8%) for a variety of reasons; lack of trust and fear. This was similar to the 71 - 84.6% reported amongst Cameroonians [26, 27], as well as the 74.3% COVID-19 vaccine rejection in Bosnia and Herzegovina [42], and higher than the 62.4 – 62.6% reported elsewhere in the Arab world and Jordan [43, 44]. The reasons for high VH were lack of trust in government’s decisions [26], people’s reluctance to get the vaccine, lack of dedicated health personnel to vaccination [16], the role of social media environments, perception of pharmaceutical industry, reliability and source of vaccine [25, 45]. Generally, there is a high rate (57.4%) of COVID-19 fear as reported in Cameroon [12], which has however dropped with time. The fear of COVID-19 has dropped for the following undocumented reasons; fear of vaccines, the assumption that COVID-19 is a simple flu, and lack of enough confidence in the vaccine development process [46]. Vaccine acceptance rates are 94.4 – 97% in Asia, 62.4% in the Arab world, 23.6% in Kuwait, 28.4% in Jordan, and 53.7 – 58.9% in Europe, and rates of 27.7% in Democratic Republic of Congo [43, 47].

### Face mask ownership/utilisation

In this study, 155 (23.3%) respondents were in households wherein all residents owned FMs, 613 (92.2%) owned FMs, 271 (40.8%) correctly wore them, and 313 (47.1%) adequately used FMs for ≥ 6 days/week. Adherence to the correct and effective use of FMs in this study was higher compared with 1.4% reported amongst Polish Health Care Workers [48]. The differences may be due to the fact that our study had a variety of occupations, while that reported in Poland was amongst Health Care Workers only.

In another study, respondents used FMs in various occasions: 95.3% for protection against COVID-19, 90.2% used FMs in public, 53% used it when entering restricted places, 45.5% when with a suspected case and 30.7% used a mask due to fear of arrest/punishment [49]. In the course of time, the use of FM too is decreasing as many, simply say COVID-19 does not exist.

Four hundred and forty (66.2%) of the respondents have very good knowledge of COVID-19 symptoms. In another study, 58.6% had moderate knowledge about COVID-19, whereas 37.2% had good knowledge [7]. The knowledge level in this study is higher compared to the 21.9% reported in Buea – Cameroon [29] and lower compared to the 68.2% reported in a multinational study [50].

### COVID-19 preventive measures

Of the five preventive measures, 570 (85.7%) of the respondents pointed out to regular hand washing, 75 (11.3%) made no mention of any of the measures, while 110 (16.5%) made mention of four of the measures. These findings were different from the adherence to barrier measures reported in Cameroon[12].

Fundamental to assessing pre-/post- COVID-19 symptoms, and ownership and utilisation rates of FMs is obtaining epidemiological data from the communities. These findings underline the need for continuous intervention programmes to enhance the prevention of the spread of COVID-19. Regular health education on COVID-19 vaccinations, and the practice of preventive measures should be encouraged. Stakeholders should try to curb vaccine hesitation by involving local scientists in the development or manufacturing process, and by dealing with conspiracies.

## Strengths and limitations

### Strengths

Field data were obtained online and from workplace/door-to-door/OPDs. The quality of the data collected was assured through pretesting of questionnaires to minimize bias as well as errors.

### Limitations

This was a cross-sectional study, representing the snapshot of the population within the study period and does not show cause and effect since the predictor and outcome variables were measured at the same time. Data was collected through anonymous self-reporting via partly online and partly workplace/door-to-door/OPD and thus there is a possibility of various types of bias; selection/double selection bias, and recall bias. Such biases can also affect some of the responses and subsequently the results of the study. Other limitations to this study were that online respondents might not have met the inclusion criteria, FMs were not categorised into surgical, air filtering respirators or simple cloth masks.

## Conclusion

In this study, we described the symptoms and severity of COVID-19 infection as well as length of hospital stay/quarantine, face mask utilisation and vaccine hesitation/acceptance amongst persons who have recovered from COVID - 19. There was a variety of symptoms with unproductive cough and fatigue as the most prevalent symptoms occurring before and after treatment. The majority of patients had no comorbidity and the commonest comorbidities were gastritis and high blood pressure. Despite the prescription of various treatment regimens, the length of stay in the hospital or quarantine was long.

Thus, the treatment of COVID-19 does not mean immediate recovery as the mild to moderate grade severity persists. Face mask availability and ownership does not mean higher utilisation. This study advocates for an intensification of COVID-19 preventive practices, as well as elaborate education on the importance of vaccination and preparation for future disease outbreaks.

## Supporting information

S1 Checklist (DOC)

S1 Appendix Survey questionnaire. (PDF)

S1 File Supplementary tables. (DOCX)

S2 File (DOCX)

S1 Data (XLSX)

## Abbreviations

$\bar{x}$: Mean
COVID-19: Coronavirus disease 2019
FM(s): Face mask(s)
SD: Standard Deviation
VH: Vaccine hesitancy
WHO: World Health Organisation
χ^2^: Chi-square
