# Supplementary material for: Face mask ownership/utilisation and COVID-19 vaccine hesitancy amongst patients recovering from COVID-19 in Cameroon: A cross-sectional study
Source: PLoS One. 2023 Jan 20;18(1):e0280269. doi: 10.1371/journal.pone.0280269 (PMC9858007; doi:10.1371/journal.pone.0280269)
Supplement: S1 Appendix — (PDF) [file pone.0280269.s002.pdf]

QUESTIONNAIRE ON PRE- AND POST-SYMPTOMS OF COVID-19

Answer all the questions by completely checking (√) in the box(es) to the side of the answer or filling the blank spaces provided.

Please this survey is for those who have tested positive for the COVID-19 virus and have undergone treatment.

Health Area:___________________________, Quarter: _____________________________

Weight (Kg):___________________________, Height (m): _____________________________

SECTION A: SOCIO-DEMOGRAPHIC DATA

1. Age: ________

2. Gender (*Tick; √, only one*): ☐ Male, ☐ Female

3. Marital status (*Tick; √, only one*): ☐ Single, ☐ Married, ☐ Widow(er), ☐ Divorced/ Separated

4. What was the highest level of schooling that you completed? (*Tick; √, only one*).

A. ☐ No formal education (NFE)

B. ☐ Primary education,

D. ☐ Secondary/High school education,

E. ☐ Tertiary education (*After high school; Bachelor, Master, PhD*).

5. What is your occupation? (*Tick; √, only one*).

A. ☐ Civil servant

B. ☐ Student

C. ☐ Business/ Private sector

D. ☐ Farming/Fishing/ CDC worker

E. ☐ Unemployed (Housewife, Apprentice, Retired)

F. ☐ Medical personnel (Nurse, Medical Dr, Med Lab Tech, Nurse Assistant)

6. How many sleeping rooms are there in your house/home? (*Please write the number of rooms, say 0, 1, 2, 3, …*) ___________

7. How many people slept in the house last night? (*Please write the number of persons, say 0, 1, 2, 3, … years old, besides the option*).

A. Persons 0 – 59 years: ___________

B. Persons 60 years and above: ___________

8. Have you ever been operated upon? (*Y/N*)

Survey Questionnaire

9. Which of these do you suffer from? (*Tick; √, all that apply*)

A. ☐ High BP/Hypertension

B. ☐ Diabetes

C. ☐ Heart/Cardiac disease

D. ☐ Chronic obstructive pulmonary disease (COPD)/Pnemonia (Asthma)

E. ☐ Hepatitis/Liver disease

F. ☐ Renal/Kidney disease

G. ☐ Gastritis

SECTION B: OWNERSHIP AND UTILISATION OF FACE MASKS

10. About how many face mask(s) do you use in one week? (*Please write the number of masks, say 0, 1, 2, 3, …*) ____________

11. If you have face mask(s), where did you acquire it (them)? (*Tick; √, all that apply*)

A. ☐ Self-made/Tailor

B. ☐ A gift from a relation

C. ☐ From the office

D. ☐ Pharmacy

E. ☐ I don't have face mask

F. ☐ Others (*Specify*): ________________________

12. How many people in the household are having face masks? (*Please write the number of persons, say 0, 1, 2, 3, … years old, besides the option*).

A. Persons 0 – 59 years: ___________

B. Persons 60 years and above: __________

13. How do you put on the mask? (*Please tick; √, in the box to the left of the option*).

A. ☐ I don't have one

B. ☐ Below my chin

C. ☐ To cover the mouth, so as to breath with my nose

D. ☐ To cover the nose, so as to breath with my mouth

E. ☐ To cover both mouth and nose

F. To cover nose, mouth, and chin

14. In the past week, how many times have you use the mask? (*Please write the number of times, say 0, 1, 2, 3, …*) ____________

Survey Questionnaire

15. If you own face mask(s) and did not use it (them) each time you were outdoors, why not? (*Tick; √, all that apply*)

A. ☐ It gives heat

B. ☐ It (They) was (were) dirty

C. ☐ I put it on, only in the presence of security forces

F. ☐ Hard to be consistent

16. How often do you change your face mask(s)? (*Tick; √, only one*)

A. ☐ After every three hours

B. ☐ After every six hours

C. ☐ I use one in one day

D. ☐ I use one in two days

E. ☐ I don't change them

17. What do you do with the mask(s) after use?

A. ☐ Discard

B. ☐ Wash and re-use

18. In your household, what is (are) the **main** method(s) used to protect against COVID19? (*Tick; √, all that apply*)

A. ☐ Regular hand washing

B. ☐ Have chlorinated water at the door

C. ☐ We don't welcome visitors

D. ☐ Keep house surroundings clean

E. ☐ I have no Corona Prevention Method

F. ☐ Others

SECTION C: PRE-COVID19 SYMPTOMS AND TREATMENT

19. Which of these are symptoms of COVID19? (*Tick; √, all that apply*)

A. ☐ Fever

A. ☐ Dry/Unproductive cough

B. ☐ Shortness of breath

C. ☐ Headache

D. ☐ Muscle aches

E. ☐ Fatigue/tiredness

F. ☐ Nausea/Vomiting

G. ☐ Loss of taste/smell

H. ☐ Sore/Itchy throat

I. ☐ Diarrhoea

Survey Questionnaire

20. Which of these are symptoms did you experience before testing positive for COVID19? (*Tick; √, all that apply*)

- **A.** ☐ Fever
- **B.** ☐ Dry/Unproductive cough
- **C.** ☐ Shortness of breath
- **D.** ☐ Headache
- **E.** ☐ Muscle aches
- **F.** ☐ Fatigue/tiredness
- **G.** ☐ Nausea/Vomiting
- **H.** ☐ Loss of taste/smell
- **I.** ☐ Sore/Itchy throat
- **J.** ☐ None of these

21. For how many days did you experience these symptoms? (*Please write the number of days, say 0, 1, 2, 3, …*) ____________

22. After testing positive, which treatment regimen were you placed on? (*Tick; √, all that apply*)

- **A.** ☐ The MOH prescribed regimen (*Chloroqine, azythromycin, paracetamol, zinc, Vit C*)
- **B.** ☐ ELIXIR COVID
- **C.** ☐ Sweetish bitter
- **D.** ☐ I was counselled to go home and quarentine myself.
- **E.** ☐ Other (*Please specify*)

23. Did you add some of your drugs? (*Y/N*)

24. For how long were you hospitalised? (*Please write the number of days, say 0, 1, 2, 3, …*) _______

25. If you were not hospitalised, for how many days did you quarantine yourself? (*Please write the number of days, say 0, 1, 2, 3, …*) _______

SECTION D: POST-COVID19 SYMPTOMS AND RECOVERY

26. Which of these are symptoms did you experience after treatment? (*Tick; √, all that apply*)

- **A.** ☐ Fever
- **B.** ☐ Unproductive cough
- **C.** ☐ Shortness of breath
- **D.** ☐ Headache
- **E.** ☐ Muscle aches/pain
- **F.** ☐ Fatigue/tiredness

27. For how many days did you experience these symptoms? (*Please write the number of days, say 0, 1, 2, 3, …*) _______

28. Will you voluntarily go for COVID-19 vaccine, if it is brought to your area? (*Y/N*)

29. If No, Why?

Thank you very much for your time and energy

Survey Questionnaire
