# Supplementary material for: Face mask ownership/utilisation and COVID-19 vaccine hesitancy amongst patients recovering from COVID-19 in Cameroon: A cross-sectional study
Source: PLoS One. 2023 Jan 20;18(1):e0280269. doi: 10.1371/journal.pone.0280269 (PMC9858007; doi:10.1371/journal.pone.0280269)
Supplement: S1 File — (DOCX) [file pone.0280269.s003.docx]

**Supplementary Tables**

**Supplementary Table 1: Knowledge of COVID-19 symptoms and FM use frequency**

| **Symptom** | **Frequency** | **Percent** |
| --- | --- | --- |
| Shortness of breath | 607 | 91.3 |
| Fatigue/tiredness | 592 | 89.0 |
| Fever | 583 | 87.7 |
| Headache | 573 | 86.2 |
| Dry/Unproductive cough | 567 | 85.3 |
| Loss of taste/smell | 535 | 80.5 |
| Sore/Itchy throat | 480 | 72.2 |
| Muscle aches | 410 | 61.7 |
| Nausea/Vomiting | 389 | 58.5 |
| Diarrhoea | 333 | 50.1 |
| Skin Rashes | 50 | 7.5 |
| **Cumulative symptoms of COVID19** |  |  |
| None | 21 | 3.2 |
| At least one symptom | 43 | 6.5 |
| At least four symptoms | 16 | 2.4 |
| Five | 52 | 7.8 |
| Six | 52 | 7.8 |
| Seven | 41 | 6.2 |
| Eight | 115 | 17.3 |
| Nine | 67 | 10.1 |
| Ten | 258 | 38.8 |
| **Graded knowledge** |  |  |
| Very Poor | 64 | 9.6 |
| Moderate | 68 | 10.2 |
| Good | 93 | 14.0 |
| Very Good | 440 | 66.2 |
| **Facemask changing frequency per day** |  |  |
| Three hourly | 56 | 8.4 |
| Six hourly | 79 | 11.9 |
| One/day | 366 | 55.0 |
| Don’t change them | 164 | 24.7 |
| **Treatment of FM after use** |  |  |
| Wash and re-use | 282 | 42.4 |
| Discard | 383 | 57.6 |
| Total | **665** | 100.0 |

**Supplementary Table 2: Household preventive measures for COVID-19**

| **Prevention measure(s) taken** | **Frequency** | **Percent** |
| --- | --- | --- |
| Regular hand washing | 570 | 85.7 |
| Keep house surroundings clean | 265 | 39.8 |
| We practice social distancing | 237 | 35.6 |
| Have chlorinated water at the door | 213 | 32.0 |
| Alcohol base hand rub | 4 | 0.6 |
| **Cummulative Measures** |  |  |
| None | 75 | 11.3 |
| At least One | 231 | 34.7 |
| At least Two | 129 | 19.4 |
| Three | 120 | 18.0 |
| Four | 110 | 16.5 |
| **Graded COVID19 household prevention measures** |  |  |
| Very Poor | 162 | 24.4 |
| Poor | 281 | 42.3 |
| Moderate | 222 | 33.4 |
| **Total** | **665** | 100.0 |

**Supplementary Table 3: Association of respondent’s characteristics with COVID-19 VH**

| **Characteristic** | *n* (%) | *p*-value | OR (95% C.I) |
| --- | --- | --- | --- |
| **Age groups (in years)** |  |  |  |
| ≤ 40/> 40 | 124/39 | 6.2E-01 | 0.9 (0.6 - 1.4) |
| **Sex** |  |  |  |
| Male/Female | 80/83 | 9.2E-01 | 1.0 (0.7 - 1.5) |
| **Marital status** |  |  |  |
| Married/Not married | 61/102 | 2.1E-01 | 0.8 (0.5 - 1.2) |
| **Education** |  |  |  |
| Secondary/Tertiary | 57/73 | 8.2E-01 | 1.0 (0.7 - 1.6) |
| Primary/Tertiary | 33/73 | 3.9E-01 | 0.7 (0.4 - 1.4) |
| **Occupation** |  |  |  |
| Student/Skilled worker | 21/64 | 4.7E-01 | 0.8 (0.4 - 1.5) |
| Unemployed/Skilled worker | 33/64 | 2.4E-01 | **1.5 (0.8 - 3.0)** |
| Unskilled/Skilled worker | 18/64 | 3.4E-01 | **1.4 (0.7 - 3.0)** |
| Business operator/Skilled worker | 27/64 | 9.5E-01 | 1.0 (0.6 - 1.7) |
| **Household size** |  |  |  |
| 5 – 9/1 – 4 | 33/130 | 4.6E-01 | **1.2 (0.7 - 2.0)** |
| **Residence** |  |  |  |
| Bamenda/Yaoundé | 16/67 | 5.1E-01 | **1.3 (0.6 - 2.5)** |
| Bafoussam/Yaoundé | 15/67 | 6.2E-01 | **1.2 (0.6 - 2.4)** |
| Buea/Yaoundé | 7/67 | 6.5E-02 | 0.4 (0.2 - 1.0) |
| Douala/Yaoundé | 58/67 | 1.2E-01 | 0.7 (0.5 - 1.1) |
