# Supplementary material for: Face mask ownership/utilisation and COVID-19 vaccine hesitancy amongst patients recovering from COVID-19 in Cameroon: A cross-sectional study
Source: PLoS One. 2023 Jan 20;18(1):e0280269. doi: 10.1371/journal.pone.0280269 (PMC9858007; doi:10.1371/journal.pone.0280269)
Supplement: S2 File — (DOCX) [file pone.0280269.s004.docx]

**Title**: Face mask Ownership/Utilisation and COVID-19 Vaccine Hesitancy Amongst Patients Recovering from COVID-19 in Cameroon: A Cross-sectional Study

**Principal** **Investigator**: Cho Frederick Nchang

**Organization**: University of Buea

**PART 1. INFORMATION SHEET**

**Introduction**

I am Cho Frederick Nchang, a Student in the Department of Biochemistry and Molecular Biology of the Faculty of Sciences in the University of Buea. Please carefully read through this document (or understand its contents as read by a witness) before accepting to participate in this study.

The aim of this study is to establish pre-/post Coronavirus Disease 2019 (COVID-19) diagnosis/treatment symptoms, ownership/utilisation of face masks, as well as vaccine hesitancy amongst patients recovering from COVID-19. This study has been cleared by the North West Regional Delegation of Public Health.

**Purpose and Background of the study**

As you probably know, COVID-19 is a pandemic as well as a major disease in Cameroon and can be transmitted from one person to another through cough, sneezing, and touch. Many approaches are being used to reduce the disease. People of all ages can be infected, with the old being the most susceptible. The Ministry of Public Health in collaboration with stakeholders has subsidised testing and drugs. Some Health Districts, have been selected for this study. We have obtained authorisation from the Regional Delegate of Health, for this study. To reduce the incidence of the infection, and to understand the ownership and utilisation of face masks in the prevention of COVID-19, our team is contacting you, in consultation with the Chief Medical Officer/and or Quarter Head, to investigate these issues.

**Information on face masks and vaccines**

Face masks and vaccines have been supplied at the various health facilities.

**Type of study**

In this study, we seek to establish the pre- and post-COVID-19 diagnosis/treatment symptoms amongst persons who suffered from COVID-19 infection, ownership and utilisation of face masks, as well as vaccine hesitancy.

**Participant selection**

We are presenting this questionnaire to you because you tested positive for COVID-19, was treated and you are recovering.

**Procedures**

I would therefore like to have your consent to participate in filling this questionnaire; this will last about 10 minutes. In the questionnaire, I will ask you some questions about yourself, the use of face masks as well as your vaccine status.

**Confidentiality**

All information related to your participation will be kept confidential and will not be revealed to anyone. Your identity will not be revealed in any reports or publications resulting from the study. The results obtained from the questionnaire will be put into a computer, but with the code numbers assigned to you. The data collected will be kept for analysis. It will be stored for some time on paper and in the computer, but may eventually be destroyed.

**Voluntary participation: right to refuse or withdraw consent / benefits**

Your participation in the study is entirely voluntary. You are not under any obligation to participate, and you have the right to refuse this invitation.

If at any time upon filling the questionnaire, you decide not to participate further, you are free to withdraw immediately, with no further discussion; this will have no adverse consequences for you.

You are not entitled to any financial benefits for participating in this study.

**Who to contact**

If you have any questions, please ask them, either now or later; through the following:

Mr Cho Frederick Nchang, Phone number: 672 788 034

This protocol has been authorised by the North West Regional Delegate for Public Health.

**PART 2. CERTIFICATE OF CONSENT**

*I have read this information in English or it has been read to me in English. I have had the opportunity to ask questions about it, and any questions that I have asked have been answered to my satisfaction. I consent voluntarily to participate in this study, and I understand that I have the right to withdraw from the study at any time without in any way affecting my rights.*

_________ _____________________________ _________________________

Place, Date Signature of Patient/ Legal Representative If representative: name, address

Thank you, Date of signature of Researcher

Cho Frederick Nchang ______/______/______

(*dd*/*mm*/*yy*)

Master Candidate, Molecular Epidemiology and Diagnostic Science

University of Buea
