# Supplementary material for: Face mask ownership/utilisation and COVID-19 vaccine hesitancy amongst patients recovering from COVID-19 in Cameroon: A cross-sectional study
Source: PLoS One. 2023 Jan 20;18(1):e0280269. doi: 10.1371/journal.pone.0280269 (PMC9858007; doi:10.1371/journal.pone.0280269)
Supplement: Attachment — Submitted filename: PONE-D-22-15424_CN.pdf [file pone.0280269.s007.pdf]

PLOS ONE

Pre- and Post-COVID-19 Diagnosis/Treatment Symptoms, Face mask Ownership/Utilisation, COVID-19 Preventive Measures, and COVID-19 Vaccine Hesitancy Amongst Patients Recovering from the COVID-19 infection in Cameroon: A Cross-Sectional Study

--Manuscript Draft--

**Manuscript Number:**	PONE-D-22-15424
**Article Type:**	Research Article
**Full Title:**	Pre- and Post-COVID-19 Diagnosis/Treatment Symptoms, Face mask Ownership/Utilisation, COVID-19 Preventive Measures, and COVID-19 Vaccine Hesitancy Amongst Patients Recovering from the COVID-19 infection in Cameroon: A Cross-Sectional Study
**Short Title:**	Pre-/Post-COVID-19 Diagnosis/Treatment Symptoms, Face mask Ownership, & COVID-19 Vaccine Hesitancy in Cameroon
**Corresponding Author:**	Frederick Nchang Cho, Master of Science<br>University of Buea<br>Buea, SW CAMEROON
**Keywords:**	Coronavirus Disease 2019; Symptoms of COVID-19; Face mask ownership/utilisation; Vaccine hesitancy; Knowledge of COVID-19 symptoms; Practice of preventive measures.
**Abstract:**	Introduction: This study aimed to establish pre-/post Coronavirus Disease 2019 (COVID-19) symptoms, ownership/utilisation of face masks (FMs), as well as vaccine hesitancy (VH) amongst patients recovering from COVID-19.<br>Methods: A cross-sectional survey was conducted from April - July and August - October 2021. Data was collected with structured self-administered questionnaires. Multinomial regression was used to determine associations between ownership/utilisation of FMs with respondents' characteristics.<br>Results: Unproductive cough and fatigue were prevalent before and after treatment. Pre-/Post COVID-19 symptoms severity ranged from asymptomatic to moderate. There was a COVID-19 VH rate of 74%. The prevalence of FM ownership and utilisation were, respectively, 92.2% and 40.8%. Factors associated with FM ownership were respondent's sex, education, and family size. The main reasons for irregular utilisation were; inability to be consistent, only used outdoors, and boredom.<br>Conclusion: The treatment of COVID-19 does not mean immediate recovery as mild to moderate grade severity still persists. Face mask availability and ownership does not mean appreciable utilisation. This study advocates for an intensification of COVID-19 preventive practices, as well as elaborate education on the importance of vaccination.
**Order of Authors:**	Frederick Nchang Cho, Master of Science
	Yayah Emerencia Ngah, MPH, M.Sc
	Andrew N Tassang, M.D
	Neh Celestina Fru, PhD
	Elad Peter Canisius Kuku, M.Sc
	Jokwi Patrick Kofon, BMLS
	Folefac Valmie Ngassam, M.Sc
	Ismaila Esa, MPH, M.Sc
	Fru Paulette Ngum, MPH, M.Sc
**Additional Information:**	
**Question**	**Response**
**Financial Disclosure**	The authors received no specific funding for this work

Powered by Editorial Manager® and ProduXion Manager® from Aries Systems Corporation

Enter a financial disclosure statement that describes the sources of funding for the work included in this submission. Review the submission guidelines for detailed requirements. View published research articles from *PLOS ONE* for specific examples.<br><br>This statement is required for submission and **will appear in the published article** if the submission is accepted. Please make sure it is accurate.<br><br>**Unfunded studies**<br>Enter: *The author(s) received no specific funding for this work.*<br><br>**Funded studies**<br>Enter a statement with the following details:<br>• Initials of the authors who received each award<br>• Grant numbers awarded to each author<br>• The full name of each funder<br>• URL of each funder website<br>• Did the sponsors or funders play any role in the study design, data collection and analysis, decision to publish, or preparation of the manuscript?<br>• **NO** - Include this sentence at the end of your statement: *The funders had no role in study design, data collection and analysis, decision to publish, or preparation of the manuscript.*<br>• **YES** - Specify the role(s) played.<br><br>* typeset	
**Competing Interests**<br><br>Use the instructions below to enter a competing interest statement for this submission. On behalf of all authors, disclose any competing interests that could be perceived to bias this work—acknowledging all financial support and any other relevant financial or non-financial competing interests.<br><br>This statement is required for submission and **will appear in the published article** if	The authors have declared no competing interests exist.

the submission is accepted. Please make sure it is accurate and that any funding sources listed in your Funding Information later in the submission form are also declared in your Financial Disclosure statement.<br><br>View published research articles from *PLOS ONE* for specific examples.<br><br>**NO authors have competing interests**<br><br>Enter: *The authors have declared that no competing interests exist.*<br><br>**Authors with competing interests**<br><br>Enter competing interest details beginning with this statement:<br><br>*I have read the journal's policy and the authors of this manuscript have the following competing interests: [insert competing interests here]*<br><br>* typeset	
**Ethics Statement**<br><br>Enter an ethics statement for this submission. This statement is required if the study involved:<br><br>• Human participants<br>• Human specimens or tissue<br>• Vertebrate animals or cephalopods<br>• Vertebrate embryos or tissues<br>• Field research<br><br>Write "N/A" if the submission does not require an ethics statement.<br><br>General guidance is provided below. Consult the submission guidelines for detailed instructions. **Make sure that all information entered here is included in the Methods section of the manuscript.**	This study was conducted in accordance with the Helsinki Declaration as well as the principles of Personal Information Protection and Electronic Documents Act, and cleared by the North West Regional Delegation of Public Health (Reference No: 95/ATT/NWR//RDPH/BRIGAD). Only respondents who consented to the study, were allowed to participate by filling and submitting the anonymous questionnaire.

Powered by Editorial Manager® and ProduXion Manager® from Aries Systems Corporation

**Format for specific study types**<br><br>**Human Subject Research (involving human participants and/or tissue)**<br>• Give the name of the institutional review board or ethics committee that approved the study<br>• Include the approval number and/or a statement indicating approval of this research<br>• Indicate the form of consent obtained (written/oral) or the reason that consent was not obtained (e.g. the data were analyzed anonymously)<br><br>**Animal Research (involving vertebrate animals, embryos or tissues)**<br>• Provide the name of the Institutional Animal Care and Use Committee (IACUC) or other relevant ethics board that reviewed the study protocol, and indicate whether they approved this research or granted a formal waiver of ethical approval<br>• Include an approval number if one was obtained<br>• If the study involved *non-human primates*, add *additional details* about animal welfare and steps taken to ameliorate suffering<br>• If anesthesia, euthanasia, or any kind of animal sacrifice is part of the study, include briefly which substances and/or methods were applied<br><br>**Field Research**<br><br>Include the following details if this study involves the collection of plant, animal, or other materials from a natural setting:<br>• Field permit number<br>• Name of the institution or relevant body that granted permission	
**Data Availability**<br><br>Authors are required to make all data underlying the findings described fully available, without restriction, and from the time of publication. PLOS allows rare exceptions to address legal and ethical concerns. See the PLOS Data Policy and FAQ for detailed information.	Yes - all data are fully available without restriction

A Data Availability Statement describing where the data can be found is required at submission. Your answers to this question constitute the Data Availability Statement and **will be published in the article**, if accepted.<br><br>**Important:** Stating 'data available on request from the author' is not sufficient. If your data are only available upon request, select 'No' for the first question and explain your exceptional situation in the text box.<br><br>Do the authors confirm that all data underlying the findings described in their manuscript are fully available without restriction?	
**Describe where the data may be found in full sentences. If you are copying our sample text, replace any instances of XXX with the appropriate details.**<br><br>• If the data are **held or will be held in a public repository**, include URLs, accession numbers or DOIs. If this information will only be available after acceptance, indicate this by ticking the box below. For example: *All XXX files are available from the XXX database (accession number(s) XXX, XXX.).*<br>• If the data are all contained **within the manuscript and/or Supporting Information files**, enter the following: *All relevant data are within the manuscript and its Supporting Information files.*<br>• If neither of these applies but you are able to provide **details of access elsewhere**, with or without limitations, please do so. For example:<br><br>*Data cannot be shared publicly because of [XXX]. Data are available from the XXX Institutional Data Access / Ethics Committee (contact via XXX) for researchers who meet the criteria for access to confidential data.*<br><br>*The data underlying the results presented in the study are available from (include the name of the third party*	The data used to support the findings of this study are included within the supplementary information file(s).

and contact information or URL).<br>• This text is appropriate if the data are owned by a third party and authors do not have permission to share the data.<br><br>* typeset	
Additional data availability information:	Tick here if your circumstances are not covered by the questions above and you need the journal’s help to make your data available.

Powered by Editorial Manager® and ProduXion Manager® from Aries Systems Corporation

Revised manuscript
Click here to access/download;Manuscript;Manuscript.docx

Pre- and Post-COVID-19 Diagnosis/Treatment Symptoms, Face mask Ownership/Utilisation, COVID-19 Preventive Measures, and COVID-19 Vaccine Hesitancy Amongst Patients Recovering from the COVID-19 infection in Cameroon: A Cross-Sectional Study

Pre- and Post-COVID-19 Diagnosis/Treatment Symptoms, Face mask Ownership, and COVID-19 Vaccine Hesitancy in Cameroon: A Cross-Sectional Study

Frederick Nchang Cho[1,2,3‡*], Yayah Emerencia Ngah[4‡], Andrew N Tassang[5,6,7], Celestina Neh Fru[7,8], Peter Canisius Kuku Elad[9,10], Patrick Kofon Jokwi[9], Valmie Ngassam Folefac[11,12], Ismaila Esa[9], Paulette Ngum Fru[13,14]

[1]Global Health Systems Solutions, Cameroon.

[2]Infectious Disease Laboratory, Faculty of Health Sciences, University of Buea, Cameroon.

[3]Central Africa Network for Tuberculosis, HIV/AIDS and Malaria (CANTAM), University of Buea, Cameroon.

[4]District Health Services Bamenda, North West Regional Delegation of Health, Ministry of Health, Cameroon.

[5]Department of Obstetrics and Gynaecology, Faculty of Health Sciences, University of Buea, Cameroon.

[6]Buea Regional Hospital Annex, Buea, Cameroon.

[7]Atlantic Medical Foundation, Mutengene, Cameroon.

[8]Department of Sociology and Anthropology, Faculty of Social and Management Sciences, University of Buea, Cameroon.

[9]Cameroon Baptist Convention Health Services, Cameroon.

[10]Department of Microbiology and Parasitology, University of Buea, Cameroon.

[11]Department of Biochemistry and Molecular Biology, Faculty of Science, University of Buea, Cameroon.

[12]Sintieh Research Academy, Cameroon.

[13]Department of Public Health and Hygiene, Faculty of Health Sciences, University of Buea, Cameroon.

[14]District Health Services Tiko, South West Regional Delegation of Health, Ministry of Health, Cameroon.

[‡]These authors contributed equally to this work.

*Correspondence should be addressed to; nchang.cho@gmail.com (FNC)

Abstract

Introduction: This study aimed to establish pre-/post Coronavirus Disease 2019 (COVID-19) symptoms, ownership/utilisation of face masks (FMs), as well as vaccine hesitancy (VH) amongst patients recovering from COVID-19.

Methods: A cross-sectional survey was conducted from April - July and August - October 2021. Data was collected with structured self-administered questionnaires. Multinomial regression was used to determine associations between ownership/utilisation of FMs with respondents' characteristics.

Results: Unproductive cough and fatigue were prevalent before and after treatment. Pre-/Post COVID-19 symptoms severity ranged from asymptomatic to moderate. There was a COVID-19 VH rate of 74%. The prevalence of FM ownership and utilisation were, respectively, 92.2% and 40.8%. Factors associated with FM ownership were respondent's sex, education, and family size. The main reasons for irregular utilisation were; inability to be consistent, only used outdoors, and boredom.

Conclusion: The treatment of COVID-19 does not mean immediate recovery as mild to moderate grade severity still persists. Face mask availability and ownership does not mean appreciable utilisation. This study advocates for an intensification of COVID-19 preventive practices, as well as elaborate education on the importance of vaccination.

Key words: Coronavirus Disease 2019; Symptoms of COVID-19; Face mask ownership/utilisation; Vaccine hesitancy; Knowledge of COVID-19 symptoms; Practice of preventive measures.

Introduction

A novel enveloped ribonucleic acid (RNA) beta (β) corona virus, Coronavirus Disease 2019 (COVID-19) has spread widely from Wuhan, causing tens of thousands of deaths, especially in patients with severe COVID-19 as from December 2019 (1–5). The coronavirus diseases vary from mild, self-limiting forms to more severe manifestations depending on the type of viruses involved (6,7). As of mid-March 2022, about 461,175,583 cases, 6,071,057 deaths, and 394,441,157 recovered cases have been reported globally (8). The impacts of the pandemic have been felt unequally around the world, with Europe and America being highly affected, as shown by overwhelmed health systems and high death tolls (9).

The Coronavirus Disease 2019 (COVID-19) is rapidly spreading across Africa, and available data indicates that it is on the rise in Uganda, Cameroon, and other African countries (10,11). A third wave of the pandemic, characterised by the delta (δ) strain of the virus, has also reached parts of Africa. The first case of COVID-19 in Cameroon was identified on the 6th of March 2020 (12) and as of 6th July 2020, 320 deaths were recorded and almost 15,000 cases were confirmed (13–15). As of the 29th of December 2021, 3,756 health personnel tested positive for COVID-19 nationwide (16), of whom two of the 45 from the South West Region of Cameroon died (17), and as of mid-March 2022, 119,322 cases, 1,926 dead, and 117,263 recovered nationwide (8). As of 28th July 2021, 285,522 people had received the first dose of the vaccine and 53,365 the second, representing 38% consumption of the received vaccines (17).

The reference and definitive diagnosis of Severe Acute Respiratory Syndrome Coronavirus-2 (SARS-CoV-2) infection is the reverse transcription polymerase chain reaction assay (rt-PCR). Chest X-ray (CXR), although not generally considered sensitive for the detection of pulmonary abnormalities in the early stage of the disease, can be a useful diagnostic tool for monitoring the

rapid progression of lung involvement in COVID-19, especially in patients admitted to intensive care units (ICUs) (18).

Many approaches have been used for the treatment of high mortality risk patients as well as other patients (19,20). The recovery of patients presenting with varying symptoms depends on treatment regimens administered (19), as well as the severity of the infection. The clinical spectrum of COVID-19 varies from asymptomatic presentation (Stage I) to moderate to severe states (Stages IIa and IIb) characterized by respiratory failure necessitating, mechanical ventilation and ICU support and those manifesting with critical clinical condition (Stage III), with mild to moderate disease occurs in approximately in 81% of cases (21).

Individuals of all ages are at risk for infection and severe disease. However, the probability of serious COVID-19 disease is higher in the old, those living in nursing homes or long-term care facilities, and those with chronic medical conditions (20).

In Cameroon, SARS-CoV-2 screening, hydroxychloroqine/azithromycin regimens and COVID-19 vaccines are free in all Health Districts while the ADSAK/ELIXIR COVID costs 20,000Fcfa (30.6€ or 36.3$) and is available in Catholic and Private Health Facilities across the Country. As of 8th July 2021, the following COVID-19 vaccines; Sinopharm BIBP, and Oxford–AstraZeneca were in Cameroon, as well as Janssen (Johnson & Johnson) donated by the United States Government, and the African Union (16). A variety of face masks (FMs) are available in Pro-pharmacies in most health facilities, local shops, tailoring shops, and street vendors. The average cost of FMs was 350Fcfa (0.53€ or 0.63$); range 200 – 500Fcfa (0.31 – 0.76€ or 0.36 – 0.91$). The majority of the population using FMs, use the locally made masks/cloth masks that costs 200Fcfa (0.31€ or 0.31$). The monthly income of the population ranges between 16.65 and 72.11 € (or 19.81 and 85.81$) (22). Conversion rates; 1€ = 655Fcfa, 1$ = 551.13Fcfa.

This study aimed to establish pre- and post-COVID-19 symptoms amongst persons who suffered from COVID-19 infection, ownership and utilisation of face masks, as well as vaccine hesitancy.

Methods

Study design and setting

A cross-sectional study was conducted from April 29th - July 4th, and August 2nd - October 30th, 2021 amongst persons who had tested positive for COVID-19. Patients who admitted having recovered from COVID-19 as confirmed by a negative control test result, were enrolled into this study. Information on pre-/post-recovery clinical symptoms were collected with the use of anonymous online as well as 'hand-filled' questionnaires (S1 Appendix).

Study population and target sample size

The study population was persons who by chance had new technology devices and could access social media platforms, and those who were at home/work place/outpatient department (OPD) of selected hospitals. The Google© questionnaire's link was distributed via social media: WhatsApp, LinkedIn, Facebook, Skype, and Instagram. It was also shared from door-to-door, work places and OPD of hospitals in the towns of Bafoussam, Douala, and Yaoundé. Being COVID-19 positive, adult (≥ 20 years of age) and consent to participate in the study constituted the inclusion criteria.

An estimated minimum sample size of 256 per town was calculated with the CDC Epi Info 7 StatCalc, and collected by purposive sampling, considering the following characteristics: an estimated Cameroon population size of 23,344,000 in 2015 (22), an assumed frequency of persons who have recovered from COVID-19 of 50%, accepted error margin of 5%, and design effect of 2.0.

Sampling method

The study was conducted during the second wave of the COVID-19 pandemic in Cameroon. Participants were approached and informed of the study objectives via social media platforms, at their work places, at their door steps and OPD of hospitals. Investigators continuously posted the questionnaire together with reminders on various platforms, regularly checked incoming responses, as well as regularly checked workplaces, homes, and OPDs to collect 'hand dropped' questionnaires.

Data collection and analysis

Data was collected with the use of anonymous self-administered online (3,4) and workplace/door-to-door/OPD questionnaires using Microsoft Office Excel and analysed with CDC Epi Info version 7.2. The questionnaire that consisted of 29 questions, aimed to collect information on: socio-demographic characteristics, ownership/utilisation of FMs, pre-/post-symptoms, and treatment of COVID-19, as well as opinions on COVID-19 vaccination. The survey instrument took approximately 10 minutes to complete. The validity of the questionnaire was confirmed by pre-testing in 10 participants who were excluded from the study. Based on the pre-test study, the format and wording of some questions were corrected and refined. Data from the 10 participants was used to assess internal consistency reliability using Cronbach's alpha (α) (23–25). The results showed adequate internal consistency reliability (with Cronbach's $\alpha = 0.72$) (23,24).

Analysis of variance (ANOVA) and Multinomial Logistic Regression were used to determine associations between hospitalisation/quarantination and ownership/utilisation of FMs with demographic characteristics. In order to control for confounders, the least significant independent covariates were excluded from the Multinomial Logistic Regression analysis. The following models were used:

Ownership of FM = $\beta_0 + \beta_1$Age + β_2Sex + β_3Marital Status + β_4Education + β_5Occupation + β_6Family size + ε, and

Household ownership of FM/Correct wearing of FM/ = $\beta_0 + \beta_1$Age + β_2Sex + β_3Marital Status + β_4Education + β_5Occupation + β_6Family size + β_7Residential area + ε. Where β_0 is a constant, β_1, β_2, β_3, ..., and β_7 are coefficients and ε is the regression error.

The significance level was set at < 0.05.

Definition of concepts/dependent variables

Severity of COVID-19: The severity grading of COVID-19 in this study consisted of pre-/post-treatment symptoms and their duration, age (≥60 years considered riskier), and comorbidities (five; on a score of 2) with a severity grading of < 20% for asymptomatic, 20 - < 40% for mild, 40 - < 80% for moderate and ≥ 80% for severe COVID-19.

Ownership of face masks: The proportion of respondents who had at least one FM, where the numerator comprised the number of respondents surveyed with at least one FM and the denominator, the total number of respondents surveyed. *Entire household ownership of FMs*; the proportion of households wherein all *de facto* members have FMs, where the numerator comprises the number of households wherein all *de facto* members own FMs and the denominator, the total number of respondents surveyed.

Utilisation of face masks: *The correct wearing of FMs*; the proportion of respondents who correctly wore FM, where the numerator comprises the number of respondents surveyed correctly wearing FMs as recommended by the WHO (26) and the denominator, the total number of respondents surveyed. *Adequate utilisation of FMs*; the proportion of respondents who wore at least six or more (≥6) FMs per week, where the numerator comprises the number of respondents surveyed adequately wearing FMs and the denominator, the total number of respondents surveyed. *Regular utilisation of FMs*; the proportion of respondents who regularly wore their FMs, where the numerator comprises the number of respondents surveyed regularly wearing FMs and the denominator, the total number of respondents surveyed.

Knowledge related to COVID-19 symptoms: Knowledge related to symptoms of COVID-19 comprised one open-ended question with the possibility of listing 10 or more options. The options were developed by considering previous studies with a similar research theme (27). The options were sorted in the form of yes or no; if the answer was yes/no, a score of '1'/'0' was accorded to the participant. Modified Bloom's cut-off points were used to judge knowledge as very poor (< 20%), poor (20 - < 40%), moderate (40 - < 60%), good (60 - < 80%), and very good (≥ 80%) (7).

Practice regarding COVID-19 prevention: Prevention practices consisted of the correct and adequate wearing of FMs, and a question on preventive measures towards COVID-19 (one correct point listed by the respondent earned a score of 1 and an omission or wrong point listed earned a score of 0). Modified Bloom's cut-off points were used to judge knowledge as very poor (< 20%), poor (20 - < 40%), moderate (40 - < 60%), good (60 - < 80%), and very good (≥ 80%)(7).

COVID-19 Vaccine hesitancy: The proportion of respondents who will not voluntarily take COVID-19 vaccine, where the numerator comprises the number of respondents who will not take the vaccine (28) and the denominator, the total number of respondents surveyed.

Ethical Consideration

This study was conducted in accordance with the Helsinki Declaration (29) as well as the principles of Personal Information Protection and Electronic Documents Act (30), and cleared by the North West Regional Delegation of Public Health (Reference №: 95/ATT/NWR//RDPH/BRIGAD). Only respondents who consented to the study, were allowed to participate by filling and submitting the anonymous questionnaire.

Strengths and limitations

Strengths: Field data were obtained online and from workplace/door-to-door/OPDs. The quality of the data collected was assured through pretesting of questionnaires to minimize bias as well as errors.

Limitations: This was a cross-sectional study, representing the snapshot of the population within the study period and does not show cause and effect since the predictor and outcome variables were measured at the same time. Data was collected through anonymous self-reporting and thus there is a possibility of bias where the respondent provides socially acceptable answers. Being a part online and part workplace/door-to-door/OPD study, there was also the possibility of selection as well as recall bias, as only those with internet devices and those at work/home/hospital were opportune to participate in the study. Such biases can also affect some of the responses and subsequently the results of the study. Another limitation to this study was that FMs were not categorised into surgical, air filtering respirators or simple cloth masks.

Results

Characteristics of Study Population

Six hundred and ninety persons were logged into our Microsoft Excel sheet, from whom 56 were dropped as outliers for age, BMI, and prolonged hospitalisation. Of the 665 persons included in this study, 2,126 household residents were counted; 1,751 (82.4%) were persons 0 – 59 years old and 375 (17.6%) were persons ≥ 60 years old. Four hundred and thirty-seven (52.2%) persons were females and 318 (47.8%) were males. The mean age of the participants was 34 years (SD 9.0, range 20 – 64). Three hundred and ninety (58.6%) were singles, 308 (45.9%) were of tertiary educational status, and 268 (40.3%) were skilled workers (Table 1).

Table 1: Characteristics of study participants

General characteristic	Subclass	Count (%)
Age groups (in years)	≤ 40	510 (76.7)
	> 40	155 (23.3)
	Mean age ($\bar{x}$ ± SD)	34.00 ± 9.00
Sex	Female	437 (52.2)
	Male	318 (47.8)
BMI (Kg/m^2)	Eutrophic	256 (38.5)
	Overweight	233 (35.0)
	Obese	176 (26.5)
	Mean BMI ($\bar{x}$ ± SD)	27.00 ± 4.00
Marital status	Not married	390 (58.6)
	Married	275 (41.4)

Education	Primary	142 (21.4)
	Secondary	218 (32.8)
	Tertiary	305 (45.9)
Occupation	Business/Private sector	118 (17.7)
	Student	97 (14.6)
	Unemployed	118 (17.7)
	Unskilled worker	64 (9.6)
	Skilled worker	268 (40.3)
Household size	1 – 4	546 (82.1)
	5 – 9	119 (17.9)
	Mean household size ($\bar{X}$ ± SD)	3.00 ± 1.00
Number of sleeping rooms	One	209 (31.4)
	Two	365 (54.9)
	More than two (3 - 5)	91 (13.7)
	Mean number of rooms ($\bar{X}$ ± SD)	1.00 ± 0.00
Comorbid condition	Gastritis	202 (30.4)
	Previously operated	89 (13.4)
	High blood pressure	108 (16.2)
	Hepatitis/Liver disease	95 (14.3)
	Diabetes	50 (7.5)
Number of comorbidities	None	202 (30.4)
	At least one	384 (57.7)
	At most two	77 (11.7)

BMI; Body Mass Index [Eutrophic (18.5 ≤ BMI ≥ 24.9), Overweight (25.0 ≤ BMI ≥ 29.9), Obese (BMI ≥ 30.0)], SD; Standard Deviation.

Clinical profile of respondents

Pre- and Post- COVID-19 diagnosis/treatment symptoms: Three hundred and ten (46.6%), 289 (43.5%), and 214 (32.2%) of the respondents presented with cough, sore throat, and tiredness, respectively (Figure 1) for a mean duration of 2.30 days (SD 2.46, range 0 – 9 days) prior to diagnosis and treatment. Fatigue/Tiredness, dry/unproductive cough, and shortness of breath were experienced for a mean duration of 3.65 days (SD 3.17, range 0 – 8) after drug administration.

Figure 1: Pre- and Post-symptoms of COVID-19

Two hundred and sixty-eight (40.3%) and 316 (47.5%) of the respondents, respectively, manifested with no symptoms of COVID-19 prior to testing positive and after treatment (Figure 1).

Severity and comorbidities: The severity of pre- and post-COVID-19 diagnosis and treatment are presented in Figure 2. The most common comorbidities were gastritis 202 (30.4%), high blood

pressure 108 (16.2%), and hepatitis and/or other liver diseases 95 (14.3%), with 202 (30.4%) having no comorbidity and 77 (11.7%) having at most two comorbidities (Table 1).

Figure 2: Severity grading for pre- and post-COVID-19 amongst age groups

Duration of hospitalisation/quarantine: The mean duration of hospitalisation and quarantine periods were, respectively, 2.95 days (SD 7.20, range 0 – 24) and 6.84 days (SD 7.00, range 0 – 28). Male respondents stayed longer in the hospital and in quarantine than females (Table 2).

Table 2: Association of hospitalisation/quarantination with respondents' characteristics; ANOVA

Variable	Subclass	Hospitalisation (*in days*)		Quarantination (*in days*)	
		$\bar{X}$ ± SD	F (*p* – value)	$\bar{X}$ ± SD	F (*p* – value)
Age (in years)	≤ 40	2.92 ± 7.20	$2.0x10^{-2}$ ($8.6x10^{-1}$)	6.70 ± 7.03	$1.1x10^{0}$ ($3.0x10^{-1}$)
	> 40	3.02 ± 7.12		7.35 ± 6.74	
Sex	Female	2.62 ± 6.84	$1.5x10^{-1}$ ($2.2x10^{-1}$)	6.81 ± 6.93	$1.2x10^{-2}$ ($9.1x10^{-1}$)
	Male	3.30 ± 7.48		6.87 ± 7.02	
BMI (kg/m^2)	Eutrophic	2.52 ± 6.79	2.8 ($2.4x10^{-1}$)	6.58 ± 6.65	$3.0x10^{-1}$ (**$5.3x10^{-2}$**)
	Overweight	3.30 ± 7.56		7.05 ± 6.71	
	Obese	3.13 ± 7.14		6.95 ± 7.74	
	Total	2.95 ± 7.20		6.84 ± 7.00	

Bold numbers are significant *p* – values. BMI; Body Mass Index [Eutrophic (18.5 ≤ BMI ≥ 24.9), Overweight (25.0 ≤ BMI ≥ 29.9), Obese (BMI ≥ 30.0)]

Care, treatment and vaccination hesitancy: Patients received either the Ministry of Health's (MOH) protocol of oral hydroxychloroquine, paracetamol, vitamin C, zinc, and azithromycin or the local remedy ADSAK/ELEXIR COVID or both. Depending on the severity and comorbidities, some patients received or added extra doses to the prescribed doses.

Five hundred and seventy-three (86.2%) of the respondents were prescribed and administered the hydroxychloroqine/Azithromycin/Paracetamol/Zinc/Vitamin C regimen, 25 (3.8%) were simply advised to go home, and 230 (34.6%) opted to supplement the MOH's regimen with the local remedy ADSAK/ELIXIR COVID (Table 3).

Table 3: Treatment regimens for respondents

Treatment	Frequency	Percent
Counselled to go home for self-quarantine	25	3.8
Hydroxychloroqine/Azithromycin/Paracetamol/Zinc/Vitamin C	573	86.2
ADSAK/ELIXIR COVID	230	34.6
**Cumulative treatment**		
None	81	12.2
At least one treatment regimen	340	51.1
Both treatment regimens	244	36.7
Supplemented medication	260	39.1

Only 165 (24.8%) of the respondents admitted that they will accept the vaccine when it is feasible, thus yielding a COVID-19 hesitancy rate of 74% (Figure 3). From multinomial regression analysis, unmarried persons, the unemployed as well as the unskilled workers, and those residing in Bamenda and Bafoussam, were more likely to deny COVID-19 vaccine when compared with their counterparts (Supplementary Table 3).

Figure 3: COVID-19 Vaccine hesitancy and reasons for hesitancy

Ownership/Utilisation and sources of face masks

Ownership/Utilisation of face masks: Of the 665 respondents sampled, 155 (23.3%) were in households wherein all residents owned FMs (Figure 4).

Two hundred and seventy-one (40.8%) of the respondents correctly wore their FMs to cover the nose, mouth, and chin, such so as to be able to breathe, while 313 (47.1%) adequately/effectively used FMs for ≥ 6 days/week (Figure 4).

Figure 4: Ownership/Utilisation of face masks

Factors associated with ownership/utilisation of face masks: Multinomial logistic regression was used to test if respondent characteristics were significantly associated with face mask ownership/utilisation. The results indicated that the predictors of the ownership of FMs by the entire household were sex (OR; 1.1, 95%C.I; 0.7 – 1.5), marital status (OR; 1.3, 95%C.I; 0.8 – 2.0), primary level education (OR; 1.3, 95%C.I; 0.6 – 2.6), students as well as business operators [(OR; 1.6, 95%C.I; 0.9 – 3.1) vs (OR; 1.3, 95%C.I; 0.7 – 2.1)] and Bamenda as well as Buea [(*p*; $<1.0\times10^{-3}$, OR; 7.6, 95%C.I; 3.8 – 15.5) vs (*p*; $<1.0\times10^{-3}$, OR; 14.6, 95%C.I; 6.5 – 33.1)] (Table 4).

Table 4: Association of respondents' characteristics with face mask ownership/utilisation

	Ownership of FM (*n* = 613)			Entire household own FM (*n* = 155)		
Characteristic	*n* (%)	*p*-value	OR (95% C.I)	*n* (%)	*p*-value	OR (95% C.I)
**Age groups (in years)**						
≤ 40/> 40	472/141	2.6x10^{-1}	1.5 (0.7 – 3.1)	110/45	8.7x10^{-1}	1.0 (0.6 – 1.8)
**Sex**						
Male/Female	286/327	**4.5x10^{-2}**	0.5 (0.3 – 1.0)	76/79	9.1x10^{-1}	1.1 (0.7 – 1.5)
**Marital status**						
Married/Not married	253/360	2.9x10^{-1}	0.7 (0.4 – 1.3)	68/87	2.4x10^{-1}	1.3 (0.8 – 2.0)
**Education**						
Secondary/Tertiary	198/283	4.1x10^{-1}	0.7 (0.4 – 1.5)	42/89	9.6x10^{-1}	1.0 (0.5 – 1.6)
Primary/Tertiary	132 (21.5)	9.7x10^{-1}	1.1 (0.4 – 3.0)	24 (15.5)	5.6x10^{-1}	1.3 (0.6 – 2.6)
**Occupation**						
Student/Skilled worker	87/244	6.2x10^{-1}	0.8 (0.3 – 1.9)	27/69	1.3x10^{-1}	1.6 (0.9 – 3.1)
Unemployed/Skilled worker	108 (17.6)	7.0x10^{-1}	0.8 (0.3 – 2.3)	16 (10.3)	4.3x10^{-1}	0.7 (0.3 – 1.6)
Unskilled/Skilled worker	61 (9.9)	4.9x10^{-1}	1.6 (0.4 – 6.5)	6 (3.9)	1.8x10^{-1}	0.5 (0.2 – 1.4)
Business operator/Skilled worker	113 (18.4)	2.1x10^{-1}	2.0 (0.7 – 4.4)	37 (23.9)	3.9x10^{-1}	1.3 (0.7 – 2.9)
**Household size**						
5 – 9/1 – 4	115/498	**4.1x10^{-2}**	**3.2** (1.1 – 9.6)	27/128	7.0x10^{-2}	0.6 (0.3 – 1.0)
**Residence**						
Bamenda/Yaoundé	-	-	-	31/44	**<1.0x10^{-3}**	**7.6** (3.8 – 15.5)
Bafoussam/Yaoundé	-	-	-	8 (5.2)	6.3x10^{-1}	0.8 (0.3 – 1.9)
Buea/Yaoundé	-	-	-	35 (22.6)	**<1.0x10^{-3}**	**14.6** (6.5 – 33.1)
Douala/Yaoundé	-	-	-	37 (23.9)	2.4x10^{-1}	0.7 (0.5 – 1.2)
	Correct wearing of FM (*n* = 271)			Adequate utilisation of FM (n 313		
**Age groups (in years)**						
≤ 40/> 40	209/62	9.9x10^{-1}	1.0 (0.7 – 1.5)	250/63	**2.2x10^{-3}**	1.9 (1.3 – 2.9)
**Sex**						
Male/Female	119/152	1.2x10^{-1}	0.8 (0.6 – 1.1)	152/161	7.1x10^{-1}	**1.1** (0.8 – 1.4)
**Marital status**						
Married/Not married	103/168	**1.3x10^{-2}**	0.6 (0.5 – 1.0)	123/190	6.1x10^{-2}	0.7 (0.5 – 1.0)
**Education**						
Secondary/Tertiary	74/133	**4.1x10^{-2}**	0.6 (0.4 – 1.0)	99/152	7.9x10^{-1}	0.9 (0.6 – 1.4)
Primary/Tertiary	64 (23.6)	9.5x10^{-1}	1.1 (0.6 – 1.8)	62 (19.8)	7.4x10^{-1}	0.9 (0.5 – 1.6)
**Occupation**						
Student/Skilled worker	35/114	5.5x10^{-1}	0.8 (0.5 – 1.4)	39/149	**5.0x10^{-4}**	0.4 (0.2 – 0.6)
Unemployed/Skilled worker	55 (20.3)	6.1x10^{-1}	1.2 (0.6 – 2.1)	55 (17.6)	8.2x10^{-2}	0.5 (0.3 – 1.1)
Unskilled/Skilled worker	25 (9.2)	9.4x10^{-1}	1.0 (0.5 – 1.9)	29 (9.3)	8.6x10^{-2}	0.5 (0.3 – 1.1)
Business operator/Skilled worker	42 (15.5)	2.9x10^{-1}	0.8 (0.5 – 1.2)	41 (13.1)	1.0x10^{-4}	0.3 (0.2 – 0.6)
**Household size**						
5 – 9/1 – 4	49/222	6.2x10^{-1}	1.1 (0.7 – 1.7)	60/253	8.7x10^{-2}	1.4 (0.9 – 2.3)
**Residence**						
Bamenda/Yaoundé	15/95	2.4x10^{-1}	0.6 (0.3 – 1.3)	19/121	5.9x10^{-2}	0.5 (0.3 – 1.0)
Bafoussam/Yaoundé	30 (1.1)	**1.1x10^{-2}**	2.3 (1.2 – 4.6)	26 (8.3)	7.1x10^{-1}	0.9 (0.4 – 1.7)
Buea/Yaoundé	19 (7.0)	9.3x10^{-1}	1.0 (0.5 – 1.9)	23 (7.4)	9.7x10^{-1}	1.0 (0.5 – 2.0)
Douala/Yaoundé	112 (41.3)	6.1x10^{-1}	1.1 (0.8 – 1.6)	124 (39.6)	4.0x10^{-1}	0.8 (0.6 – 1.2)

OR = Odds Ratio; C.I. = Confidence Interval; **Boldface** numbers indicate significant *p* values.

Respondents with the primary level of education (*p*; 9.5x10^{-1}, OR; 1.1, 95% C.I; 0.6 – 1.8) were more likely to correctly use FMs than those with the tertiary educational status. More females had FMs than males and were less likely to correctly wear them compared to males (*p*; 1.2x10^{-1}, OR; 0.8, 95% C.I; 0.6 – 1.1) (Table 4).

The reasons advanced for the irregular use of FMs from respondents' perspective were: 'it is hard to be consistent' (39.7%), 'I wear them whenever I am going outdoors' (28.7%), and 'it is boring/uncomfortable' (27.2%) (Figure 5).

Figure 5: Reasons for irregular use of face masks

Source of face masks: Respondents either purchased FMs, 58.8% or obtained them freely from the office/workplace 35.8%, and a gift from a relationship 35.2%, or self-made 17.3% (Table 5).

Table 5: Sources of face masks

Source of face masks	Frequency	Percent
A gift from a relationship	234	35.2
Purchased from the shop/Pharmacy	238	35.8
From the office/Workplace	391	58.8
Self-made/Tailor	115	17.3

Knowledge of COVID-19 symptoms and household preventive measures against it

Knowledge of COVID-19 symptoms: Of the 10 symptoms anticipated, 21 (3.2%) of the respondents indicated ignorance, while 258 (38.8%) were informed on all 10 (Supplementary Table 1). Four hundred and forty (66.2%) of the respondents have very good knowledge of COVID-19 symptoms, with a knowledge score of ≥80%. Those who were 40 years or less (p; 1.7×10^{-2}, OR; 1.7, 95% C.I; 1.1 - 2.6), unskilled workers as well as business operators [(OR; 1.3, 95% C.I; 0.6 - 2.7) vs (OR; 1.2, 95% C.I; 0.7 - 2.0)], and those residing in Buea as well as Douala [(OR; 1.3, 95% C.I; 0.6 - 2.9) vs (OR; 1.1, 95% C.I; 0.7 - 1.6)], were more likely to have very good knowledge of COVID-19 symptoms when compared with their counterparts (Table 6).

Household preventive measures of COVID-19: Of the five preventive measures, 570 (85.7%) of the respondents pointed out to regular hand washing, 75 (11.3%) made no mention of any of the measures, while 110 (16.5%) made mention of four of the measures (Supplementary Table 2).

Table 6: Association of respondent's characteristics with knowledge of COVID-19 symptoms/household preventive measures of COVID-19

Respondent's Characteristic	VG Knowledge of symptoms (n = 440)			Moderate HH preventive measures (n = 222)		
**Age groups (in years)**	*n* (%)	*p*-value	OR (95% C.I)	*n* (%)	*p*-value	OR (95% C.I)
≤ 40/> 40	344/96	**1.7x10^{-2}**	1.7 (1.1 - 2.6)	175/47	1.2x10^{-1}	1.4 (0.9 - 2.2)
**Sex**						
Male/Female	200/240	3.6x10^{-1}	0.8 (0.6 - 1.2)	96/126	1.9x10^{-1}	0.8 (0.6 - 1.1)
**Marital status**						
Married/Not married	185/255	8.0x10^{-1}	0.9 (0.6 - 1.4)	89/133	2.7x10^{-1}	0.8 (0.6 - 1.1)
**Education**						
Secondary/Tertiary	142/205	5.0x10^{-1}	0.9 (0.6 - 1.3)	72/104	5.8x10^{-1}	0.8 (0.6 - 1.3)
Primary/Tertiary	93 (21.1)	1.9x10^{-1}	0.6 (0.4 - 1.2)	46 (20.7)	4.9x10^{-1}	0.8 (0.4 - 1.5)
**Occupation**						
Student/Skilled worker	53/182	**3.8x10^{-3}**	0.5 (0.3 - 0.8)	24/96	**8.0x10^{-3}**	0.5 (0.3 - 0.8)
Unemployed/Skilled worker	75 (17.1)	6.0x10^{-1}	0.8 (0.5 - 1.6)	36 (16.2)	2.0x10^{-1}	0.7 (0.4 - 1.2)
Unskilled/Skilled worker	47 (10.7)	4.8x10^{-1}	1.3 (0.6 - 2.7)	24 (10.8)	8.4x10^{-1}	0.9 (0.5 - 1.8)
Business operator/Skilled worker	83 (18.9)	5.1x10^{-1}	1.2 (0.7 - 2.0)	42 (18.9)	8.5x10^{-1}	0.9 (0.6 - 1.5)
**Household size**						
5 – 9/1 – 4	76/364	6.0x10^{-1}	0.9 (0.6 - 1.4)	40/182	5.1x10^{-1}	1.7 (0.7 - 1.8)
**Residence**						
Bamenda/Yaoundé	21/167	**4.0x10^{-4}**	0.3 (0.2 - 0.6)	9/87	**1.0x10^{-2}**	0.4 (0.2 - 0.8)
Bafoussam/Yaoundé	31 (7.0)	**4.5x10^{-2}**	0.5 (0.3 - 1.0)	14 (6.3)	7.8x10^{-2}	0.5 (0.3 - 1.1)
Buea/Yaoundé	35 (7.9)	4.6x10^{-1}	1.3 (0.6 - 2.9)	13 (5.9)	1.7x10^{-1}	0.6 (0.3 - 1.2)
Douala/Yaoundé	186 (42.3)	6.8x10^{-1}	1.1 (0.7 - 1.6)	99 (44.6)	7.4x10^{-1}	1.1 (0.7 - 1.6)

OR = Odds Ratio; C.I. = Confidence Interval; VG = Very Good; HH = Household; **Boldface** numbers indicate significant *p* values.

Two hundred and twenty-two (8.4%) implemented moderate (≥50%) preventive measures against COVID-19. Those who were 40 years or less (OR; 1.4, 95%C.I; 0.9 - 2.2), students (*p*; 8x10^{-3}, OR; 0.5, 95%C.I; 0.3 - 0.8), and those residing in Bamenda (*p*; 1x10^{-2}, OR; 0.4, 95%C.I; 0.2 - 0.8), were more likely to moderately implement COVID-19 preventive measures when compare with those older than 40 years, skilled workers, and those residing in Yaoundé respectively (Table 6).

Discussion

Our study adds to the description of COVID-19 in Cameroon where there is a large gap in the literature. Our study revealed that; pre-/post symptoms were dry cough and tiredness, with sore throat and headache occurring more before diagnosis/treatment while shortness of breath persisted after treatment. Gastritis, high blood pressure, and hepatitis were the underlying comorbidities of COVID-19 amongst respondents. Majority of the respondents owned FMs, few households owned FMs, while the use of FMs was generally low in all aspects. Many respondents feared COVID-19 vaccines leading to a very high VH rate.

Cough, tiredness, and headache occurred in patients before and after treatment. A study from Ethiopia proved that these symptoms were confirmed amongst COVID-19 cases, with other common symptoms being sore throat (31). Fever, fatigue, and cough, however, remain the most common symptoms in many cohort studies (32,33).

Disease severity ranged from asymptomatic (41.5% vs 43.9%) to mild (28.6% vs 18.5%) to moderate (29.9% vs 37.6%) before and after treatment. This was similar to findings reported in Ethiopia (31). Nearly two-thirds of the patients suffered from at least one comorbidity (57.7%), of which gastritis was the most common (30.4%). This was similar to the 29% reported in Ethiopia (31), and different from the findings of another study (34), in which it was asserted that the most common comorbidities were obesity, diabetes and hypertension. These differences may be as a result of differences in study population and study design.

The mean duration of hospitalisation and quarantine period were, respectively, 2.95 days (SD 7.20, range 0 – 24) and 6.84 days (SD 7.00, range 0 – 28). This was lower compared with the 13.5 – 42 days (SD, 9.7) reported in studies elsewhere (31–33). Recovery, however, depends on the severity of the infection as well as comorbidities. Severity seems to have dropped with time, thus leading to both little hospitalisation and quarantine periods.

Five hundred and seventy-three (86.2%) of the respondents were prescribed and administered the hydroxychloroqine/Azithromycin/Paracetamol/Zinc/Vitamin C regimen, 3.8% were counselled to go home. In a similar study, all patients received a treatment protocol with oral chloroquine, paracetamol, vitamin C, zinc, amoxicillin combined with clavulanic acid, and azithromycin. Further, some patients received anticoagulants, corticosteroids, or intravenous antibiotics, with 15% of confirmed cases undergoing non-invasive ventilation (31).

In this study, vaccine hesitancy (VH) was 74.0% (vaccine acceptance of 24.8%) for a variety of reasons; lack of trust and fear. This was similar to the 71 - 84.6% reported amongst Cameroonians

(31,35), as well as the 74.3% COVID-19 vaccine rejection in Bosnia and Herzegovina (36), and higher than the 62.4 – 62.6% reported elsewhere in the Arab world and Jordan (37,38). The reasons for high VH were lack of trust in government's decisions (35), people's reluctance to get the vaccine, lack of dedicated health personnel to vaccination (16), the role of social media environments, perception of pharmaceutical industry, reliability and source of vaccine (39,40). Generally, there is a high rate (57.4%) of COVID-19 fear as reported in Cameroon (12), which has however dropped with time. The fear of COVID-19 has dropped for the following undocumented reasons; fear of vaccines, the assumption that COVID-19 is a simple flu, and lack of enough confidence in the vaccine development process (41). Vaccine acceptance rates are 94.4 – 97% in Asia, 62.4% in the Arab world, 23.6% in Kuwait, 28.4% in Jordan, and 53.7 – 58.9% in Europe, and rates of 27.7% in Democratic Republic of Congo (37,42).

In this study, 155 (23.3%) respondents were in households wherein all residents owned FMs, 613 (92.2%) owned FMs, 271 (40.8%) correctly wore them, and 313 (47.1%) adequately used FMs for ≥ 6 days/week. Adherence to the correct and effective use of FMs in this study was higher compared with 1.4% reported amongst Polish Health Care Workers (43). The differences may be due to the fact that our study had a variety of occupations, while that reported in Poland was amongst Health Care Workers only.

In another study, respondents used FMs in various occasions: 95.3% for protection against COVID-19, 90.2% used FMs in public, 53% used it when entering restricted places, 45.5% when with a suspected case and 30.7% used a mask due to fear of arrest/punishment (44). In the course of time, the use of FM too is decreasing as many, simply say COVID-19 does not exist.

Four hundred and forty (66.2%) of the respondents have very good knowledge of COVID-19 symptoms. In another study, 58.6% had moderate knowledge about COVID-19, whereas 37.2% had good knowledge (7). The knowledge level in this study is higher compared to the 21.9%

reported in Buea – Cameroon (45) and lower compared to the 68.2% reported in a multinational study (46).

Of the five preventive measures, 570 (85.7%) of the respondents pointed out to regular hand washing, 75 (11.3%) made no mention of any of the measures, while 110 (16.5%) made mention of four of the measures.

Fundamental to assessing pre-/post- COVID-19 symptoms, and ownership and utilisation rates of FMs is obtaining epidemiological data from the communities. These findings underline the need for continuous intervention programmes to enhance the prevention of the spread of COVID-19. Regular health education on COVID-19 vaccinations, and the practice of preventive measures should be encouraged. Stakeholders should try to curb vaccine hesitation by involving local scientists in the development or manufacturing process, and by dealing with conspiracies.

Conclusion

The treatment of COVID-19 does not mean immediate recovery as the mild to moderate grade severity persists. Face mask availability and ownership does not mean higher utilisation. This study advocates for an intensification of COVID-19 preventive practices, as well as elaborate education on the importance of vaccination and preparation for future disease outbreaks.

Abbreviations

$\bar{x}$: Mean, COVID-19: Coronavirus disease 2019, FM(s): Face mask(s), SD: Standard Deviation, VH: Vaccine hesitancy, WHO: World Health Organisation, χ^2: Chi-square.

Declarations

Ethics approval and consent to participate

Participation in the study was voluntary, and all participants freely consented to the study, by accepting to submit the anonymous questionnaire.

Competing interests

The authors declare that they have no competing interests.

Data availability and Supplementary Information

The data used to support the findings of this study are included in the study and within the supplementary information file (S2 Appendix).

Supplementary Information

S1 Appendix. Survey Questionnaire (PDF)

S2 Appendix. Supplementary Tables (MS Word)

S3 Appendix. Online version of questionnaire (https://forms.gle/Wevui24dn5DEVE3A9)

Acknowledgments

The authors are thankful to the respondents who participated in this survey and to the survey teams; especially those of Douala and Yaoundé.

Authors' contributions

Conceptualization: FNC, YEN.

Data cleaning: YEN, PKJ.

Formal analysis: FNC.

Methodology: FNC, YEN, PCEK, VNF.

Project administration: FNC, IE, PNF.

Resources: ANT, CNF, IE.

Supervision: FNC, YEN, ANT, CNF, PNF.

Validation: All Authors.

Writing – original draft/review & editing: FNC, YEN, PCEK, PKJ.

References

1. Shang Y, Liu T, Wei Y, Li J, Shao L, Liu M, et al. Scoring systems for predicting mortality for severe patients with COVID-19. eClinicalMedicine [Internet]. 2020 Jul 1 [cited 2022 Apr 8];24. Available from: https://www.thelancet.com/journals/eclinm/article/PIIS2589-5370(20)30170-X/fulltext

2. Zhang C, Qin L, Li K, Wang Q, Zhao Y, Xu B, et al. A Novel Scoring System for Prediction of Disease Severity in COVID-19. Front Cell Infect Microbiol. 2020;10:318.

3. Lee M, Kang BA, You M. Knowledge, attitudes, and practices (KAP) toward COVID-19: a cross-sectional study in South Korea. BMC Public Health. 2021 Feb 5;21(1):295.

4. Habib MA, Dayyab FM, Iliyasu G, Habib AG. Knowledge, attitude and practice survey of COVID-19 pandemic in Northern Nigeria. PLoS ONE [Internet]. 2021 Jan 14 [cited 2022 Apr 8];16(1):e0245176. Available from: https://www.ncbi.nlm.nih.gov/pmc/articles/PMC7808653/

5. Velavan TP, Meyer CG. The COVID-19 epidemic. Trop Med Int Health [Internet]. 2020 Mar [cited 2022 Apr 8];25(3):278–80. Available from: https://www.ncbi.nlm.nih.gov/pmc/articles/PMC7169770/

6. Cui J, Li F, Shi ZL. Origin and evolution of pathogenic coronaviruses. Nat Rev Microbiol. 2019 Mar;17(3):181–92.

7. Desalegn Z, Deyessa N, Teka B, Shiferaw W, Hailemariam D, Addissie A, et al. COVID-19 and the public response: Knowledge, attitude and practice of the public in mitigating the pandemic in Addis Ababa, Ethiopia. PloS One. 2021;16(1):e0244780.

8. Tvalchrelidze A, Silagadze A. COVID-19 Coronavirus Pandemic Influence on Crude Oil Prices [Internet]. 2022 [cited 2022 Mar 15]. Available from: https://www.worldometers.info/coronavirus/#countries

9. WHO. WHO Report: Coronavirus Disease 2019 (COVID-19) Situation Report-74. World Health Organisation; 2020.

10. Okello G, Izudi J, Teguzirigwa S, Kakinda A, Van Hal G. Findings of a Cross-Sectional Survey on Knowledge, Attitudes, and Practices about COVID-19 in Uganda: Implications for Public Health Prevention and Control Measures. BioMed Res Int. 2020;2020:5917378.

11. Judson SD, Njabo KY, Torimiro JN. Regional vulnerability for COVID-19 in Cameroon. Pan Afr Med J. 2020;37(Suppl 1):16.

12. Siewe Fodjo JN, Ngarka L, Njamnshi WY, Nfor LN, Mengnjo MK, Mendo EL, et al. COVID-19 Preventive Behaviours in Cameroon: A Six-Month Online National Survey. Int J Environ Res Public Health. 2021 Mar 4;18(5):2554.

13. Béché E. Cameroonian responses to COVID-19 in the education sector: Exposing an inadequate education system. Int Rev Educ Int Z Erzieh Rev Int Pedagog. 2020;66(5–6):755–75.

14. Akumbu PW. Legitimizing the development and use of Cameroon's national languages: Lessons from COVID-19. J Cameroon Acad Sci [Internet]. 2020 Aug 4 [cited 2022 Apr 10];15(3):193–206. Available from: https://www.ajol.info/index.php/jcas/article/view/198309

15. Nguemdjo U, Meno F, Dongfack A, Ventelou B. Simulating the progression of the COVID-19 disease in Cameroon using SIR models. PloS One. 2020;15(8):e0237832.

16. MOH. Cameroon: COVID 19 Emergency Situation Report No. 21 - 1 November to 31 December 2021 - Cameroon [Internet]. ReliefWeb. 2021 [cited 2022 Apr 10]. Available from: https://reliefweb.int/report/cameroon/cameroon-covid-19-emergency-situation-report-no-21-1-november-31-december-2021

17. MOH. Cameroon's Ministry of Health; Coronavirus Disease 19 (COVID-19) Outbreak Situation Report No 13. 2020.

18. Wasilewski PG, Mruk B, Mazur S, Półtorak-Szymczak G, Sklinda K, Walecki J. COVID-19 severity scoring systems in radiological imaging – a review. Pol J Radiol [Internet]. 2020 Jul 17 [cited 2022 Apr 10];85:e361–8. Available from: https://www.ncbi.nlm.nih.gov/pmc/articles/PMC7425223/

19. Sodani P, Mucci L, Girolimetti R, Tedesco S, Monaco F, Campanozzi D, et al. Successful recovery from COVID-19 pneumonia after receiving baricitinib, tocilizumab, and remdesivir. A case report: Review of treatments and clinical role of computed tomography analysis. Respir Med Case Rep [Internet]. 2020 Jun 27 [cited 2022 Apr 10];31:101115. Available from: https://www.ncbi.nlm.nih.gov/pmc/articles/PMC7320265/

20. NIH. Coronavirus Disease 2019 (COVID-19) Treatment Guidelines. In: Health NIo, editor. National Institutes of Health. USA: National Institutes of Health; 2021.

21. Vinod N. Identifying patterns in COVID-19: Morbidity, recovery and the aftermath. Int J Clin Virol. 2020;4(1):056–64.

22. NIS. 2nd survey on the monitoring of public expenditures and the level of recipients' satisfaction in the education and health sectors in Cameroon (PETS2)," in Health Component. Yaoundé: National Institute of Statistics; 2010. 1–116 p.

23. Tavakol M, Dennick R. Making sense of Cronbach's alpha. Int J Med Educ. 2011;2:53–5.

24. Al‐ Osail AM, Al‐ Sheikh MA, Al‐ Osail EM, Al‐ Ghamdi MA, Al‐ Hawas AM, Al‐ Bahussain AS, et al. Is Cronbach's alpha sufficient for assessing the reliability of the OSCE for an internal medicine course? BMC Res Notes. 2015;2(582):6.

25. Bujang MA, Omar ED, Baharum NA. A Review on Sample Size Determination for Cronbach's Alpha Test: A Simple Guide for Researchers. Malays J Med Sci. 2018;25(6):85–99.

26. WHO. WHO updated guidance on the use of masks [Internet]. World Health Organisation; 2020 [cited 2022 Mar 20]. Available from: https://www.who.int/docs/default-source/coronaviruse/risk-comms-updates/update-30-use-of-masks.pdf?sfvrsn=eeb24c14_2

27. Alnasser AHA, Al-Tawfiq JA, Al-Kalif MSH, Shahadah RFB, Almuqati KSA, Al-Sulaiman BSA, et al. Public Knowledge, Attitudes, and Practice towards COVID-19 Pandemic in Saudi Arabia: A Web-Based Cross-Sectional Survey. Med Sci Basel Switz. 2021 Feb 16;9(1):11.

28. Thomson A, Robinson K, Vallée-Tourangeau G. The 5As: A practical taxonomy for the determinants of vaccine uptake. Vaccine. 2016 Feb 17;34(8):1018–24.

29. Shrestha B, Dunn L. The Declaration of Helsinki on Medical Research involving Human Subjects: A Review of Seventh Revision. J Nepal Health Res Counc. 2020 Jan 21;17(4):548–52.

30. Gupta S. Ethical Issues in Designing Internet-Based Research: Recommendations for Good Practice. J Res Pract [Internet]. 2017 [cited 2022 Apr 10];13(2):D1–D1. Available from: http://jrp.icaap.org/index.php/jrp/article/view/576

31. Mbarga NF, Epee E, Mbarga M, Ouamba P, Nanda H, Nkengni A, et al. Clinical profile and factors associated with COVID-19 in Yaoundé, Cameroon: A prospective cohort study. PLOS ONE [Internet]. 2021 May 12 [cited 2022 Apr 10];16(5):e0251504. Available from: https://journals.plos.org/plosone/article?id=10.1371/journal.pone.0251504

32. Sharma R, Agarwal M, Gupta M, Somendra S, Saxena SK. Clinical Characteristics and Differential Clinical Diagnosis of Novel Coronavirus Disease 2019 (COVID-19). In: Saxena SK, editor. Coronavirus Disease 2019 (COVID-19): Epidemiology, Pathogenesis, Diagnosis, and Therapeutics [Internet]. Singapore: Springer; 2020 [cited 2022 Apr 10]. p. 55–70. Available from: https://doi.org/10.1007/978-981-15-4814-7_6

33. Carfi A, Bernabei R, Landi F, Gemelli Against C-P-ACSG. Persistent Symptoms in Patients After Acute COVID-19. JAMA. 2020;324(6):603–5.

34. Boutin S, Hildebrand D, Boulant S, Kreuter M, Rüter J, Pallerla SR, et al. Host factors facilitating SARS-CoV-2 virus infection and replication in the lungs. Cell Mol Life Sci [Internet]. 2021 [cited 2022 Apr 10];78(16):5953–76. Available from: https://www.ncbi.nlm.nih.gov/pmc/articles/PMC8256233/

35. Andrew T, Tassang N, Thierry T, Cho FN, Fru PN. Vaccination Hesitancy: The Case of Cervical Cancer Vaccination in Fako Division, Cameroon. Int J Trop Dis Health. 2021 Jun 16;

36. Fojnica A, Osmanovic A, Đuzic N, Fejzic A, Mekic E, Gromilic Z, et al. COVID-19 vaccine acceptance and rejection in an adult population in Bosnia and Herzegovina. PLOS ONE [Internet]. 2022 Feb 28 [cited 2022 May 5];17(2):e0264754. Available from: https://journals.plos.org/plosone/article?id=10.1371/journal.pone.0264754

37. Kaadan MI, Abdulkarim J, Chaar M, Zayegh O, Keblawi MA. Determinants of COVID-19 vaccine acceptance in the Arab world: a cross-sectional study. Glob Health Res Policy. 2021 Jul 12;6(1):23.

38. El-Elimat T, AbuAlSamen MM, Almomani BA, Al-Sawalha NA, Alali FQ. Acceptance and attitudes toward COVID-19 vaccines: A cross-sectional study from Jordan. PLoS ONE [Internet]. 2021 Apr 23 [cited 2022 May 3];16(4):e0250555. Available from: https://www.ncbi.nlm.nih.gov/pmc/articles/PMC8064595/

39. Dinga JN, Sinda LK, Titanji VPK. Assessment of Vaccine Hesitancy to a COVID-19 Vaccine in Cameroonian Adults and Its Global Implication. Vaccines [Internet]. 2021 Feb 19 [cited 2022 Apr 5];9(2):175. Available from: https://www.ncbi.nlm.nih.gov/pmc/articles/PMC7922050/

40. Sharma K, Koirala A, Nicolopoulos K, Chiu C, Wood N, Britton PN. Vaccines for COVID-19: Where do we stand in 2021? Paediatr Respir Rev [Internet]. 2021 Sep [cited 2022 Apr 11];39:22–31. Available from: https://www.ncbi.nlm.nih.gov/pmc/articles/PMC8274273/

41. Lavigne SE. Vaccine hesitancy: Root causes and possible solutions. Can J Dent Hyg [Internet]. [cited 2022 May 2];55(2):79–82. Available from: https://www.ncbi.nlm.nih.gov/pmc/articles/PMC8219067/

42. Troiano G, Nardi A. Vaccine hesitancy in the era of COVID-19. Public Health [Internet]. 2021 May [cited 2022 Apr 11];194:245–51. Available from: https://www.ncbi.nlm.nih.gov/pmc/articles/PMC7931735/

43. Reszke R, Matusiak Ł, Krajewski PK, Szepietowska M, Białynicki-Birula R, Szepietowski JC. The Utilization of Protective Face Masks among Polish Healthcare Workers during COVID-19 Pandemic: Do We Pass the Exam? Int J Environ Res Public Health [Internet]. 2021 Jan [cited 2022 Apr 11];18(2):841. Available from: https://www.ncbi.nlm.nih.gov/pmc/articles/PMC7835780/

44. Ogoina D. COVID-19: The Need for Rational Use of Face Masks in Nigeria. Am J Trop Med Hyg. 2020 Jul;103(1):33–4.

45. Nicholas T, Mandaah FV, Esemu SN, Vanessa ABT, Gilchrist KTD, Vanessa LF, et al. COVID-19 knowledge, attitudes and practices in a conflict affected area of the South West Region of Cameroon. Pan Afr Med J. 2020;35(Suppl 2):34.

46. Masoud AT, Zaazouee MS, Elsayed SM, Ragab KM, Kamal EM, Alnasser YT, et al. KAP-COVIDGLOBAL: a multinational survey of the levels and determinants of public knowledge, attitudes and practices towards COVID-19. BMJ Open [Internet]. 2021 Feb 1 [cited 2022 Apr 12];11(2):e043971. Available from: https://bmjopen.bmj.com/content/11/2/e043971

Fig5

Click here to access/download;Figure;Fig5.png

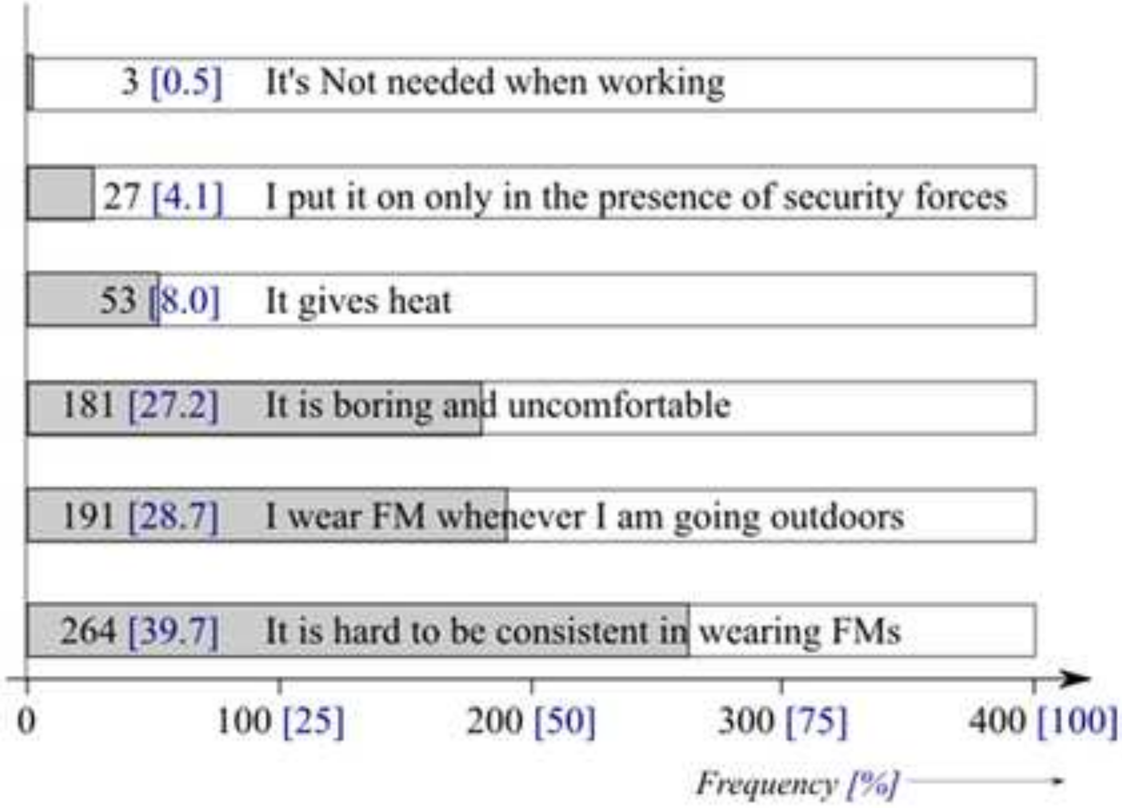

Fig4

Click here to access/download;Figure;Fig4.png

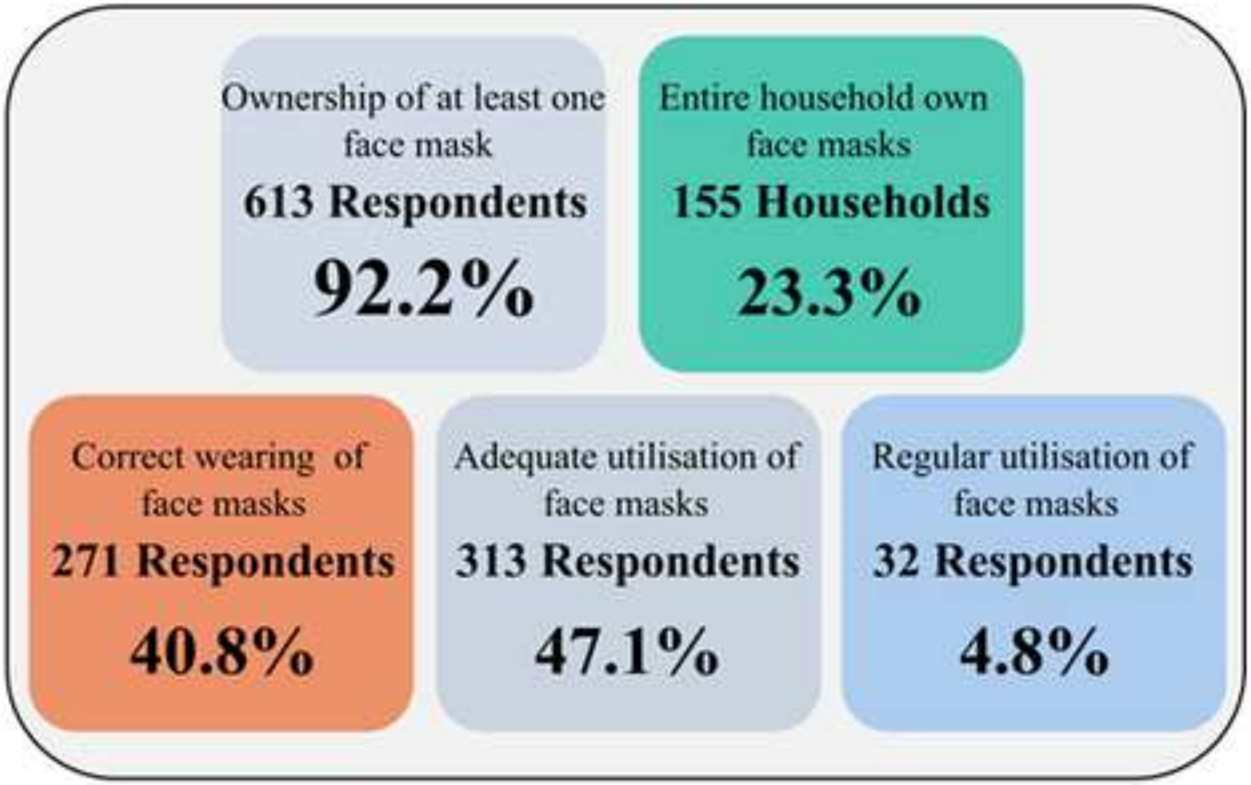

Fig3

Click here to access/download;Figure;Fig3.png

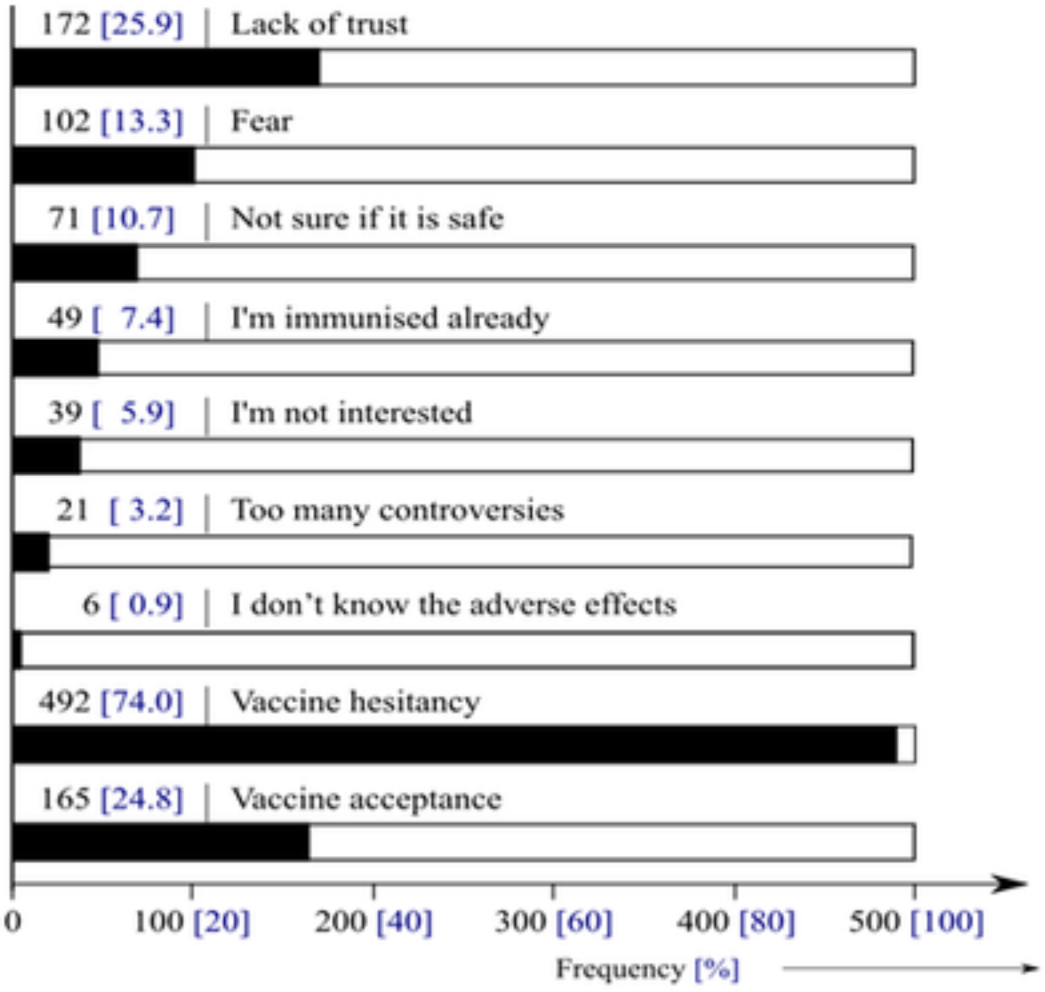

Fig2

Click here to access/download;Figure;Fig2.png

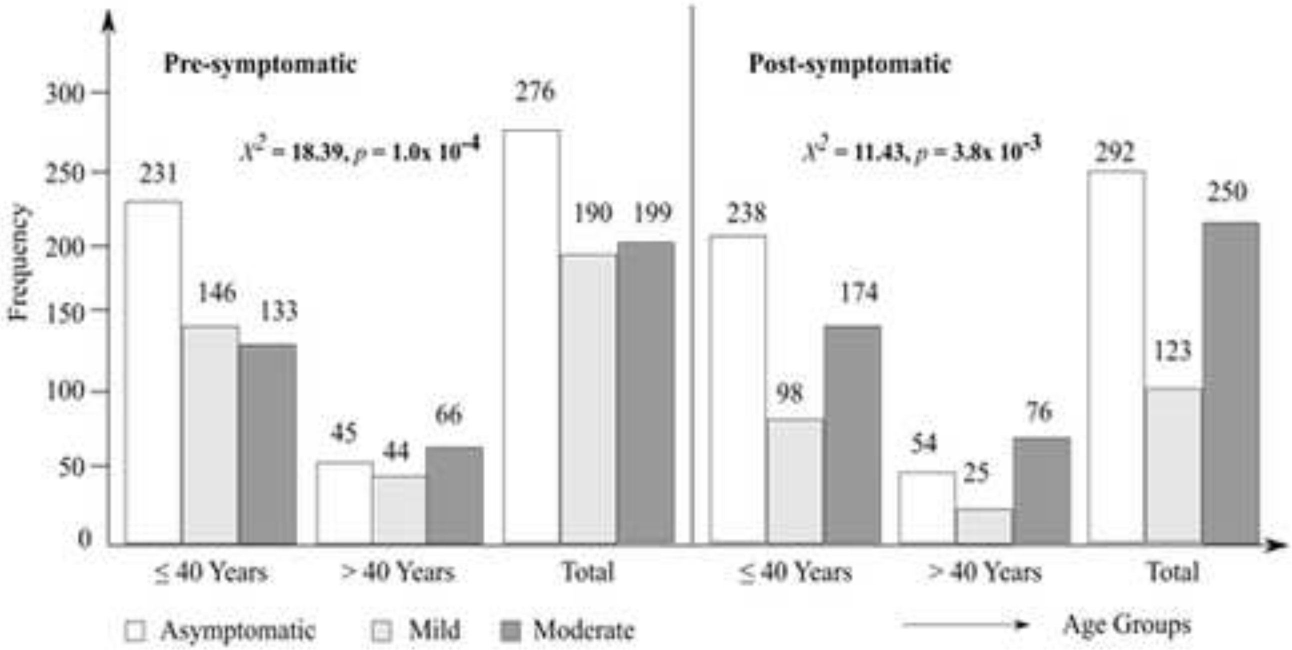

Fig1 Click here to access/download;Figure;Fig1.png

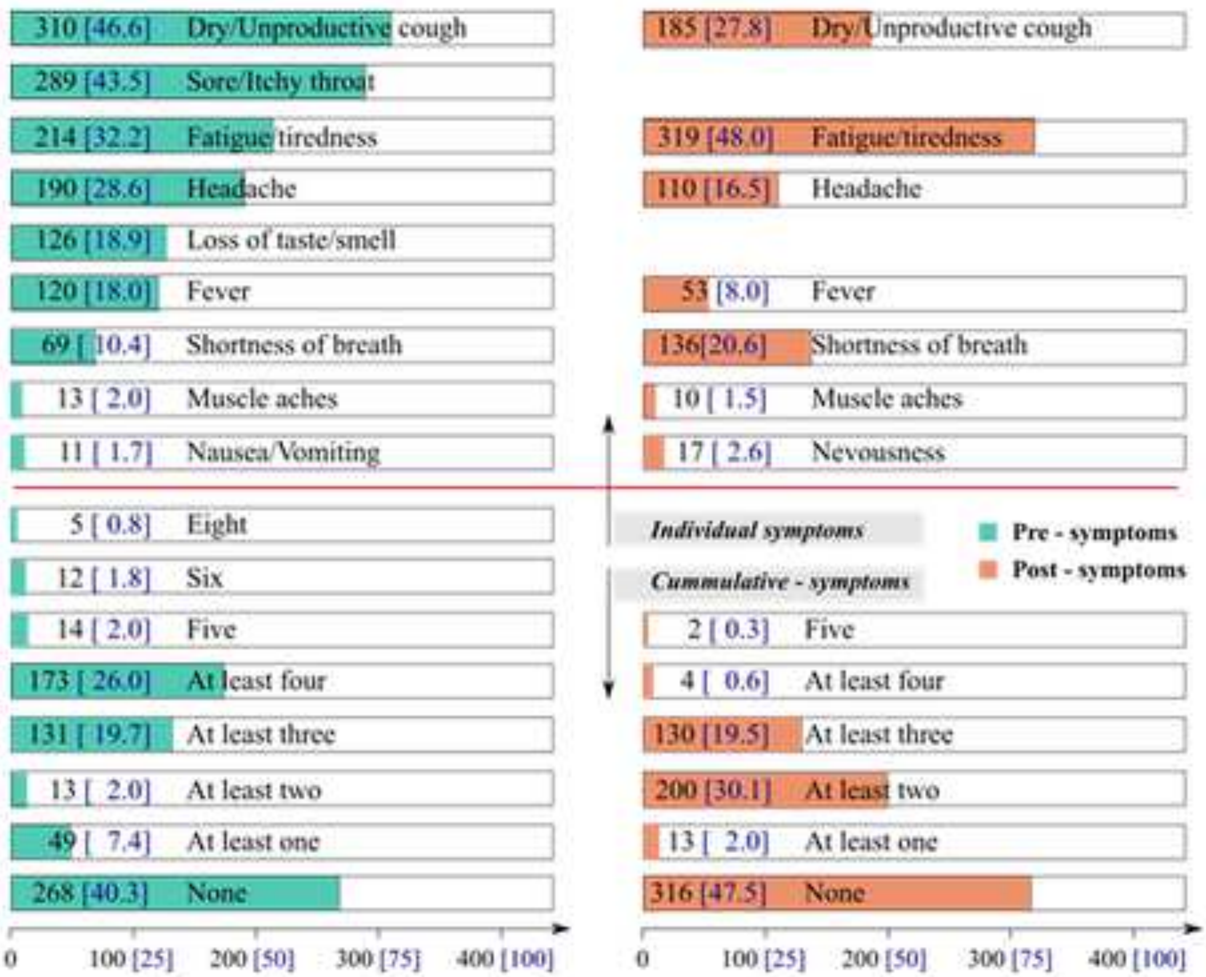

Click here to access/download
Supporting Information
Ethical Clearance.pdf

Click here to access/download
Supporting Information
S2. Supplementary Tables.docx

Click here to access/download
Supporting Information
S1 Appendix. Survey Questionnaire (PDF).pdf

Infectious Disease Laboratory,
Faculty of Health Sciences,
University of Buea
Buea – Cameroon
Sunday, 05 June 2022

The Academic Editor,
Plos One

Dear Sir,

Response to Reviewers

I am delighted once more to enclose herewith as requested, a revised version of manuscript: PONE-D-21-26364R2, with title; **Pre- and Post-COVID-19 Diagnosis/Treatment Symptoms, Face mask Ownership/Utilisation, COVID-19 Preventive Measures, and COVID-19 Vaccine Hesitancy Amongst Patients Recovering from the COVID-19 infection in Cameroon: A Cross-Sectional Study,** for re-evaluation, and possible acceptance for publication.

We heartily appreciate the views and recommendations of the reviewers. We have done our utmost best to objectively answer the questions and issues raised.

"After going through the review comments on your second revision, I feel still a lot of improvements needs to be done (which includes a suggested modification of your **study design and methodology**). **I would be happy to consider a substantially revised version of the work as a new submission provided you are ready to undertake the additional work"**.

> *In addition to collecting data through social media platforms, we also carried out door-to-door/office/OPDs administration of questionnaires. Thus, we took time off to gather questionnaires from the various field workers and entered into Microsoft Excel for analysis with Epi Info 7.*

Introduction
Please update paragraphs 1 and 2 (to actual epidemiology).

> *This was an earlier request which was adjusted and answered thus, 'This has been updated as requested; lines 62 – 98 of the **Manuscript**'.*

Study Design and Methodology

I feel still a lot of improvements needs to be done (which includes a suggested modification of your **study design and methodology**).

> *We have worked on the design as well as the entire Methods section, to include field work as well as, definition of vaccine hesitancy.*

REVIEW COMMENTS TO THE AUTHOR
Reviewer #1:

Thank you for your comments. I still consider the sample **size is too small for the design of your study**. The authors have not taken into consideration that this sample size may not be representative of the intended population of the study.

Initially, we worked only with the web-based data and sample size. Data for field-based work has been added and the sample size augmented to 665; mostly from the towns of Douala and Yaoundé.

Reviewer #2: This web-based survey conducted in Cameroon aimed to establish the pre/post COVID-19 symptoms, and ownership/utilization of facemasks as well as vaccine hesitancy amongst patients recovering from COVID-19.
[1] A weakness of the study is that no guidelines for online surveys, such as CHERRIES, were used. The authors could then explain how some of the CHERRIES criteria were applied to the present study, such as ensuring participant confidentiality and data security. See: Eysenbach, G. (2004). Improving the quality of Web surveys: the Checklist for Reporting Results of Internet E-Surveys (CHERRIES). Journal of medical Internet research, 6(3), e34.

We carried out with field work from August – October 2021; meeting respondents at their work places, Out Patient Departments, or at their home.

[2] Page 7, line 113. At the end of the introduction it is stated that "The aim of this study was to establish pre- and post-COVID-19 symptoms amongst persons who suffered the infection, as well as the ownership and utilisation of FMs". However, the objective stated in the abstract seems to be more comprehensive and includes hesitancy to vaccination.

COVID-19 vaccine hesitancy is still an issue and we have tried to clarify it the more.

[3] Page 7, line 117. The study is identified as a cross-sectional cohort study. In page 10, line 197, the authors claim that "this was a cross-sectional study". Why do the authors define the study's design as cohort?

This error has been sorted and corrected.

[4] Page 8, line 130. The sample size was calculated as if the purpose of the study was to estimate the prevalence of COVID-19. This needs to be revised, as this is not the purpose of the article.

This has been modified on the assumption that 50% of those who suffered from COVID-19 recovered.

[5] Page 8, line 140. R i386 is a sub-architecture of R software used for 32-bit builds. It would be clearer for the reader to write that R software was used than to write that "386 Statistics" was used.

The data has been re-analysed, with Epi Info 7.2.

[6] Page 8, line 152. "In order to control for confounders, only independent covariates whose Chi square ($\chi2$) p – values were < 0.05 were included in the Multinomial Logistic Regression analysis". This is not a valid strategy for handling confounds, and is widely criticized by many authors, as listed below. This must to be reviewed.
Sun, G. W., Shook, T. L., & Kay, G. L. (1996). Inappropriate use of bivariable analysis to screen risk factors for use in multivariable analysis. Journal of clinical epidemiology, 49(8), 907-916.
Heinze, G., Wallisch, C., & Dunkler, D. (2018). Variable selection-a review and

recommendations for the practicing statistician. Biometrical journal, 60(3), 431-449.
Heinze, G., & Dunkler, D. (2017). Five myths about variable selection. Transplant International, 30, 6-10.
Greenland, S. (1989). Modeling and variable selection in epidemiologic analysis. American Journal of Public Health, 79(3), 340-349.

The data has been re-analysed, and the necessary corrections effected accordingly.

[7] Page 9, line 154. This sentence is not technically correct: "A statistically significant association was declared at < 0.05". It would be better written in this way: The significance level was set at 0.05.

This has been corrected as advised.

[8] Page 10, line 177. "Modified Bloom's cut-off points were used to judge knowledge as…". What is the modified Bloom cut? Should this article be cited?: Bloom BS. Learning for mastery. Instruction and curriculum. Regional Education Laboratory for the Carolinas and Virginia, topical papers and reprints, number 1. Eval Comment 1968;1(2):12.

Bloom's taxonomy is a set of hierarchical models used for classification, depending on the objectives of the classification. Modified Bloom's cut is the modification of the Bloom's taxonomy into a "five" or "four" Likert scale. In our case, we used a five-scale and latter modified into a two-scale.

We did not invent the modified cuts, we learned from another study and thought it wise to acknowledge by citing and referencing.

[9] Results. Please avoid the use of the plus and minus symbol to denote mean and standard deviation. The notation "mean (SD)" should be used throughout instead of "mean plus/minus SD," since this is not a confidence interval. In other words, the SD should be indicated in parentheses. For example, change "The mean age (x ± SD) of participants was 36.61 ± 10.32 years (range 18 – 63)" to "The mean age of participants was 36.6 years (SD 10.3, range 18 – 63)".

All such results have been corrected as advised.

[10] Sufficient information should be supplied to allow readers to judge whether any assumptions necessary for the validity of statistical approaches (e.g., residuals are normally distributed, homoscedasticity) have been verified.

This has been sorted out.

[11] Table 2. Is the duration measured in days?

Yes, the duration is in days.

[12] Table 4. Change O.R to OR and C.I to CI.
[13] There is no sense in showing in graphs the relative and absolute frequencies simultaneously, on the same scale.

[12 - 13]; *All of these have been corrected as advised.*

Reviewer #3: Unfortunately, I was not aware of the fact that this version is already the second revision of the paper when accepting the manuscript – in my opinion, in this stage, it is quite hard to evaluate or estimate the improvement of the manuscript throughout the whole revision process.
However, I have some general comments/suggestions.
- The introduction is quite comprehensive; I would suggest a shorter introduction with a more contextual background leading to clear objectives.
- Concerning clear objectives; the aim according to the introduction is as follows: ".. to establish pre- and post-COVID-19 symptoms amongst persons who suffered the infection, as well as the ownership and utilization of FMs." Vaccine hesitancy is missing (see abstract).

The VH has been included in the abstract.

- Soundness: I expect a summary of the main findings (answers to aims) in the first paragraph of the discussion; in the current version, the severity of the infection as well as comorbidities (both not mentioned in aims) are mentioned first, followed by symptoms (which should be mentioned first + add face masks and vaccine hesitancy).

Immense thanks for this pointer, we have sorted this out "Our study revealed that; pre-/post symptoms…".

- The 'highlights section' does not reflect the highlights of the paper; furthermore, the second 'highlight' describes the method ("… are investigated") without mentioning the findings.

The highlights have been removed completely.

- Important limitations are not discussed: "the population was persons who by chance had new technology devices an could access social media platforms" which reflects a very selected sample.

Data for respondents met at home, work place or OPD of the hospital has been included; and the limitations adjusted to contain effects on outcomes.

- for interpretation of the results: what is exactly meant with 'before' (pre) and 'after' (post) COVID? How many days, weeks, months after COVID (on average)?

'Before' (pre) and 'after' (post); in our context, refers to, before being tested positive, and after treatment.

Other corrections and additions

Other corrections included Grammar and spell checks and the modification of some of the figures.

Declarations

The authors received no specific funding for this work.

The authors have declared that no competing interests exist.

We shall be grateful for your kindness in accepting our manuscript for publication.

Yours Sincerely,

Frederick Nchang Cho
