# Supplementary material for: Face mask ownership/utilisation and COVID-19 vaccine hesitancy amongst patients recovering from COVID-19 in Cameroon: A cross-sectional study
Source: PLoS One. 2023 Jan 20;18(1):e0280269. doi: 10.1371/journal.pone.0280269 (PMC9858007; doi:10.1371/journal.pone.0280269)
Supplement: Attachment — Submitted filename: Response to Reviewers.docx [file pone.0280269.s006.docx]

Infectious Disease Laboratory,

Faculty of Health Sciences,

University of Buea

Buea – Cameroon

Sunday, 05 June 2022

The Academic Editor,

Plos One

Dear **Sir,**

**Response to Reviewers**

I am delighted once more to enclose herewith as requested, a revised version of manuscript: PONE-D-21-26364R2, with title; **Pre- and Post-COVID-19 Diagnosis/Treatment Symptoms, Face mask Ownership/Utilisation, COVID-19 Preventive Measures, and COVID-19 Vaccine Hesitancy Amongst Patients Recovering from the COVID-19 infection in Cameroon: A Cross-Sectional Study,** for re-evaluation, and possible acceptance for publication.

We heartily appreciate the views and recommendations of the reviewers. We have done our utmost best to objectively answer the questions and issues raised.

“After going through the review comments on your second revision, I feel still a lot of improvements needs to be done (which includes a suggested modification of your **study design and methodology**). **I would be happy to consider a substantially revised version of the work as a new submission provided you are ready to undertake the additional work”**.

*In addition to collecting data through social media platforms, we also carried out door-to-door/office/OPDs administration of questionnaires. Thus, we took time off to gather questionnaires from the various field workers and entered into Microsoft Excel for analysis with Epi Info 7*.

**Introduction**
Please update paragraphs 1 and 2 (to actual epidemiology).

*This was an earlier request which was adjusted and answered thus, ‘This has been updated as requested; lines 62 – 98 of the* ***Manuscript****’*.

**Study Design and Methodology**

I feel still a lot of improvements needs to be done (which includes a suggested modification of your **study design and methodology**).

*We have worked on the design as well as the entire Methods section, to include field work as well as, definition of vaccine hesitancy*.

**REVIEW COMMENTS TO THE AUTHOR
Reviewer #1**:

Thank you for your comments. I still consider the sample **size is too small for the design of your study**. The authors have not taken into consideration that this sample size may not be representative of the intended population of the study.

*Initially, we worked only with the web-based data and sample size. Data for field-based work has been added and the sample size augmented to 665; mostly from the towns of Douala and Yaoundé.*

**Reviewer #2**: This web-based survey conducted in Cameroon aimed to establish the pre/post COVID-19 symptoms, and ownership/utilization of facemasks as well as vaccine hesitancy amongst patients recovering from COVID-19.
[1] A weakness of the study is that no guidelines for online surveys, such as CHERRIES, were used. The authors could then explain how some of the CHERRIES criteria were applied to the present study, such as ensuring participant confidentiality and data security. See: Eysenbach, G. (2004). Improving the quality of Web surveys: the Checklist for Reporting Results of Internet E-Surveys (CHERRIES). Journal of medical Internet research, 6(3), e34.

*We carried out with field work from August – October 2021; meeting respondents at their work places, Out Patient Departments, or at their home*.

[2] Page 7, line 113. At the end of the introduction it is stated that “The aim of this study was to establish pre- and post-COVID-19 symptoms amongst persons who suffered the infection, as well as the ownership and utilisation of FMs”. However, the objective stated in the abstract seems to be more comprehensive and includes hesitancy to vaccination.

*COVID-19 vaccine hesitancy is still an issue and we have tried to clarify it the more*.

[3] Page 7, line 117. The study is identified as a cross-sectional cohort study. In page 10, line 197, the authors claim that “this was a cross-sectional study”. Why do the authors define the study’s design as cohort?

*This error has been sorted and corrected*.

[4] Page 8, line 130. The sample size was calculated as if the purpose of the study was to estimate the prevalence of COVID-19. This needs to be revised, as this is not the purpose of the article.

*This has been modified on the assumption that 50% of those who suffered from COVID-19 recovered*.

[5] Page 8, line 140. R i386 is a sub-architecture of R software used for 32-bit builds. It would be clearer for the reader to write that R software was used than to write that "386 Statistics" was used.

*The data has been re-analysed, with Epi Info 7.2*.

[6] Page 8, line 152. “In order to control for confounders, only independent covariates whose Chi square (χ2) p – values were < 0.05 were included in the Multinomial Logistic Regression analysis”. This is not a valid strategy for handling confounds, and is widely criticized by many authors, as listed below. This must to be reviewed.
Sun, G. W., Shook, T. L., & Kay, G. L. (1996). Inappropriate use of bivariable analysis to screen risk factors for use in multivariable analysis. Journal of clinical epidemiology, 49(8), 907-916.
Heinze, G., Wallisch, C., & Dunkler, D. (2018). Variable selection-a review and recommendations for the practicing statistician. Biometrical journal, 60(3), 431-449.
Heinze, G., & Dunkler, D. (2017). Five myths about variable selection. Transplant International, 30, 6-10.
Greenland, S. (1989). Modeling and variable selection in epidemiologic analysis. American Journal of Public Health, 79(3), 340-349.

*The data has been re-analysed, and the necessary corrections effected accordingly*.

[7] Page 9, line 154. This sentence is not technically correct: “A statistically significant association was declared at < 0.05”. It would be better written in this way: The significance level was set at 0.05.

*This has been corrected as advised*.

[8] Page 10, line 177. “Modified Bloom’s cut-off points were used to judge knowledge as…”. What is the modified Bloom cut? Should this article be cited?: Bloom BS. Learning for mastery. Instruction and curriculum. Regional Education Laboratory for the Carolinas and Virginia, topical papers and reprints, number 1. Eval Comment 1968;1(2):12.

*Bloom’s taxonomy is a set of hierarchical models used for classification, depending on the objectives of the classification. Modified Bloom’s cut is the modification of the Bloom’s taxonomy into a “five” or “four” Likert scale. In our case, we used a five-scale and latter modified into a two-scale.*

*We did not invent the modified cuts, we learned from another study and thought it wise to acknowledge by citing and referencing*.

[9] Results. Please avoid the use of the plus and minus symbol to denote mean and standard deviation. The notation “mean (SD)” should be used throughout instead of “mean plus/minus SD,” since this is not a confidence interval. In other words, the SD should be indicated in parentheses. For example, change “The mean age (x ± SD) of participants was 36.61 ± 10.32 years (range 18 – 63)” to “The mean age of participants was 36.6 years (SD 10.3, range 18 – 63)”.

*All such results have been corrected as advised*.

[10] Sufficient information should be supplied to allow readers to judge whether any assumptions necessary for the validity of statistical approaches (e.g., residuals are normally distributed, homoscedasticity) have been verified.

*This has been sorted out*.

[11] Table 2. Is the duration measured in days?

*Yes, the duration is in days*.

[12] Table 4. Change O.R to OR and C.I to CI.
[13] There is no sense in showing in graphs the relative and absolute frequencies simultaneously, on the same scale.

[12 - 13]; *All of these have been corrected as advised*.

**Reviewer #3**: Unfortunately, I was not aware of the fact that this version is already the second revision of the paper when accepting the manuscript – in my opinion, in this stage, it is quite hard to evaluate or estimate the improvement of the manuscript throughout the whole revision process.
However, I have some general comments/suggestions.
- The introduction is quite comprehensive; I would suggest a shorter introduction with a more contextual background leading to clear objectives.
- Concerning clear objectives; the aim according to the introduction is as follows: “.. to establish pre- and post-COVID-19 symptoms amongst persons who suffered the infection, as well as the ownership and utilization of FMs.” Vaccine hesitancy is missing (see abstract).

*The VH has been included in the abstract*.

- Soundness: I expect a summary of the main findings (answers to aims) in the first paragraph of the discussion; in the current version, the severity of the infection as well as comorbidities (both not mentioned in aims) are mentioned first, followed by symptoms (which should be mentioned first + add face masks and vaccine hesitancy).

*Immense thanks for this pointer, we have sorted this out “Our study revealed that; pre-/post symptoms…”*.

- The ‘highlights section’ does not reflect the highlights of the paper; furthermore, the second ‘highlight’ describes the method (“… are investigated”) without mentioning the findings.

*The highlights have been removed completely*.

- Important limitations are not discussed: “the population was persons who by chance had new technology devices an could access social media platforms” which reflects a very selected sample.

*Data for respondents met at home, work place or OPD of the hospital has been included; and the limitations adjusted to contain effects on outcomes*.

- for interpretation of the results: what is exactly meant with 'before' (pre) and 'after' (post) COVID? How many days, weeks, months after COVID (on average)?

*‘Before’ (pre) and ‘after’ (post); in our context, refers to, before being tested positive, and after treatment*.

**Other corrections and additions**

*Other corrections included Grammar and spell checks and the modification of some of the figures.*

**Declarations**

*The authors received no specific funding for this work.*

*The authors have declared that no competing interests exist.*

*We shall be grateful for your kindness in accepting our manuscript for publication*.

Yours Sincerely,

Frederick Nchang Cho
