# Supplementary material for: Face mask ownership/utilisation and COVID-19 vaccine hesitancy amongst patients recovering from COVID-19 in Cameroon: A cross-sectional study
Source: PLoS One. 2023 Jan 20;18(1):e0280269. doi: 10.1371/journal.pone.0280269 (PMC9858007; doi:10.1371/journal.pone.0280269)
Supplement: Attachment — Submitted filename: Response to Reviewers.docx [file pone.0280269.s008.docx]

Infectious Disease Laboratory,

Faculty of Health Sciences,

University of Buea

Buea – Cameroon

Friday, 30 September 2022

The Academic Editor,

Plos One

Dear **Sir,**

**Response to Reviewers**

I am delighted once more to enclose herewith as requested, a revised version of manuscript: PONE-D-22-15424; **Pre- and Post-COVID-19 Diagnosis/Treatment Symptoms, Face mask Ownership/Utilisation, COVID-19 Preventive Measures, and COVID-19 Vaccine Hesitancy Amongst Patients Recovering from the COVID-19 infection in Cameroon: A Cross-Sectional Study**, for re-evaluation, and possible acceptance for publication.

We wholeheartedly appreciate the views and recommendations of the reviewers. We have done our utmost best to objectively answer the questions and concerns raised. We have also read through with the hope of carrying out slight modifications and weeding out some errors.

Thus, we are submitting as requested, the following:

*A marked-up copy of manuscript:* ***Revised Manuscript with Track Changes****.*

*An unmarked version of revised manuscript without tracked changes:* ***Manuscript****.*

*The Journal Requirements: formatting Title/Authors/Affiliations and Main body, were also reviewed once more.*

Please provide additional details regarding participant consent. In the ethics statement in the Methods and online submission information, please ensure that you have specified what type you obtained (for instance, written or verbal, and if verbal, how it was documented and witnessed). If your study included minors, state whether you obtained consent from parents or guardians. If the need for consent was waived by the ethics committee, please include this information.

*This was sorted out*

**GENERAL COMMENTS FROM THE PORTABLE DOCUMENT FORMAT (PDF)**

**Affiliations**

*CANTAM is not actually a structure in the University, but it sponsors projects in the University. However, it has been dropped and rather acknowledged*.

**Abstract**

*A severity grading scale, was established the methods section and used to grade the severity of the symptoms.*

*All other concerns raised have been addressed*.

**Introduction**

*This has been sorted*.

**Methods**

How did you ensure that those tested in April – July, were not retested in August?

*The various localities/OPDs/Offices were mapped out and visited serially*.

How was the correct wearing of FMs assessed?

*Question 13 of the questionnaire was used for this purpose*.

**Results**

Not possible for number of persons to be lower than number of households?

*The number of persons interviewed was the number of respondents, but was different from the number of residents in the household*.

**FIRST REVIEWERS COMMENTS AND RECOMMENDATIONS TO THE AUTHOR**

Line 37, Your study period is April - July, and August - October 2021 but you however present 2022 data in lines 58 and 69. Kindly review.

*In the earliest submission, the reviewers recommended that the literature should be updated. However, the statement has been re-written to contain the current recommendation*

In line 99 where you mention the aim of the study, you don't talk about Diagnosis/treatment symptoms but emphasize pre and post COVID 19 Symptoms. It is important to be uniform in the presentation of information to ease comprehension.

*This has been sorted and corrected*

The introduction section does not clearly reveal the rationale of your study. Do well to bring out the rationale of this study in the introduction section.

*We are supposing that the aim of the study, is synonymous to the rationale of the study. However, the rationale has been included and spelled out.*

Your study population and setting section not very clear, kindly make this section clearer. Line 109, How did you ensure participants targeted online through various social media platforms met all your inclusion criteria (i.e. infection history, actual current location, and country (your questionnaire doesn't capture information on the town, region, or city), and met the age requirements).

*The study setting has been clarified.*

*The limitations did indicate possibility of shortcomings*

*The questionnaire had Health Area, and Quarter, thus a capture of the town. See the limitations section for inclusion criteria.*

Line 121, Kindly clarify the sampling methods used. what is your rationale behind the selection of particular participants?

*The convenience sampling technique was implored wherein participants were approached and informed of the study objectives via social media platforms, at their work places, at their door steps and OPD of hospitals.*

Since you collected data both physically and through Social media, it will be interesting to present analyses for respondents by channel of response (physical and social Media), as this might have some correlation with outcomes.

*We thought of this; our first submission was based on the web-based respondents, which was rejected for varying reasons. Thus, we opted to pool the results together to obtain significant outcomes.*

Line 237, Figure 3 showing analysis of vaccine hesitancy is missing.

*Please vaccine hesitancy (VH) is there; second bar from the bottom.*

Line 332 - 333, You mention vaccine hesitance was for a variety of reasons and go ahead to site just two reasons. Good elaboration on the various objectives in your discussion section. I recommend you do the same for Vaccine hesitancy

*The two reasons advanced, were the most prevalent. Discussions too have been sorted out as requested.*

**SECOND REVIEWERS COMMENTS AND RECOMMENDATIONS TO THE AUTHOR**

1. The topic is interesting but the author has raised a lot of concepts that he could not full address. To talk about Pre- and Post-COVID-19 Diagnosis, Treatment, Symptoms, Face mask Ownership/Utilisation, COVID-19 Preventive Measures, and COVID-19 Vaccine Hesitancy is too much that can be full addressed by your finding. consider reducing the scope of your topic.

*We raised four concepts; Pre- and Post-COVID-19 Diagnosis/Treatment Symptoms, COVID-19 Vaccine Hesitancy, Face mask Ownership/Utilisation, and COVID-19 Preventive Measures, and al the findings were presented in the results section as well as discussed with sub-headings in the discussion section*

2. Your background doesn't showcase the problem you are addressing and the figures presented are outdated.

*We brought these on the advice of the previous reviewers. In the present review, Reviewer 1 says we worked in 2021 and cited 2022 figures. With the background differences, we have agreed to moderate the data.*

3. Your interpretation of OR are not correct. When your confidence interval crosses 1 its means there is no relationship.

*Immense appreciation for this pointer as found in tables 4 and 6, which we have sorted for correction. Usually, there are associations, which are of no significance. However, we also used p – values to assess the significance of associations.*

4. All concepts handle under your study should be clearly discussed and probably under different paragraphs

*We did raise the following concepts:*

*Pre- and Post-COVID-19 Diagnosis/Treatment Symptoms*

*COVID-19 Vaccine Hesitancy*

*Face mask Ownership/Utilisation*

*COVID-19 Preventive Measures*

*The above concepts were discussed under different paragraphs. However, we have included sub-headings for clarity.*

*We are grateful for your acceptance to review our manuscript*.

Yours Sincerely,

Frederick Nchang Cho
