# Supplementary material for: Face mask ownership/utilisation and COVID-19 vaccine hesitancy amongst patients recovering from COVID-19 in Cameroon: A cross-sectional study
Source: PLoS One. 2023 Jan 20;18(1):e0280269. doi: 10.1371/journal.pone.0280269 (PMC9858007; doi:10.1371/journal.pone.0280269)
Supplement: Attachment — Submitted filename: Response to Review.docx [file pone.0280269.s009.docx]

Infectious Disease Laboratory,

Faculty of Health Sciences,

University of Buea

Buea – Cameroon

Sunday, 30 October 2022

The Academic Editor,

Plos One

Dear **Sir,**

**Response to Reviewers**

I am delighted once more to enclose herewith as requested, a revised version of manuscript: PONE-D-22-15424R1; **Face mask Ownership/Utilisation and COVID-19 Vaccine Hesitancy Amongst Patients Recovering from COVID-19 in Cameroon: A Cross-sectional Study**, for re-evaluation, and possible acceptance for publication.

We wholeheartedly appreciate the views and recommendations of the reviewers. We have once more done our utmost best to objectively answer the questions and concerns raised. We have also read through the manuscript with the hope of carrying out slight modifications in order to render consistency with adjustments made from the reviewers’ recommendations.

Thus, we are submitting as requested, the following:

*A marked-up copy of manuscript:* ***Revised Manuscript with Track Changes****.*

*An unmarked version of revised manuscript without tracked changes:* ***Manuscript****.*

*The Journal Requirements: formatting Title/Authors/Affiliations and Main body, were also reviewed once more.*

**CONCERNS RAISED BY REVIEWER #1**

This article is now fit for publishing as all the concerns raised have been addressed by the authors have.

*Not applicable*

**CONCERNS RAISED BY REVIEWER #2**

1. The author did not response to my suggestion of reshaping the topic to make it more attractive and better support the finding. It will be good for him to provide clear justification why the topic should be maintained as it is.

*The topic has been reshaped after a deep reflection as follow,*

***Long topic****: Face mask Ownership/Utilisation and COVID-19 Vaccine Hesitancy Amongst Patients Recovering from COVID-19 in Cameroon: A Cross-sectional Study*

***Short topic****: Face mask Ownership/Utilisation and Vaccine Hesitancy Amongst Patients Recovering from COVID-19 in Cameroon*.

2. Author did not rephrase the sentence “symptoms severity range from asymptomatic to moderate” in the abstract. Asymptomatic means no symptom. Kindly indicate the symptoms to ease understanding of which was severe and which was mild

*Thank you for this; the sentence now reads; Pre-/Post COVID-19 symptoms severity ranged from mild to moderate*

3. Line 103: The author has not explained how he ensured that those surveyed from April to July 4 were not surveyed in August since the study was conducted in two sections. What is a repeated survey?

*This has been sorted and addressed with the ensuing sentence.*

*Those who consented to fill the questionnaire, were cautioned not to fill it if they had earlier filled a similar online questionnaire*.

*Further, the limitations section, also defines the possible types of bias especially double selection.*

*The study was not a repeated study*.

4. Line 161, Author talk about wearing of MF correctly but he has not explained how he assessed this for those who participate in the survey by responding online questionnaire.

*This was a self-administered questionnaire; the wearing of FM was described on the questionnaire Q13*: How do you put on the mask? (Please tick; √, in the box to the left of the option).
*A. ☐ I don’t have one
B. ☐ Below my chin
C. ☐ To cover the mouth, so as to breath with my nose
D. ☐ To cover the nose, so as to breath with my mouth
E.* ***☐ To cover both mouth and nose*** *F. ☐ To cover nose, mouth, and chin*

*Respondents who choose the WHO recommended method of wearing FM, were assumed to wear their FMs correctly. Further, the Limitations section of the manuscript admits that there is a likelihood of recall bias.*

5. Line 206 and 207: Please check this out it is not possible for the number of persons (665) enrolled to be lower than the number households (2,126).

*Sir, you are very correct. We interviewed one person per household; one who had suffered from COVID-19 and got treated. In the questionnaire, we enquired the number of persons living in the household.*

*Thus, the statement is, 665 persons/household included in the study, 2,126 de facto household residents counted in the 665 households.*

*This means 1,461 (2,126 - 665) persons were exposed*.

6. Line 261-264 the use of more likely indicate association, which is not consistent with your p-values. The still need to be addressed. Same with line 270.

*This use of this ‘more likely’, is coming up in the presentation of results of Supplementary Table 3, Table 4, and* *6**. All these tables, deal with the determination of Odds Ratio (OR); the odds of a parameter of the independent variable to affect the explanatory variable. Ratios, deal with likelihoods/probabilities, are actually calculated through the log likelihood equation, and should be reported as such. However, the OR can also be reported in terms of odds and so, some of the ‘more likely’, were rephrased*.

**GENERAL CONCERNS**

*This reference, was removed because it was duplicated*

*WHO. Cameroon: WHO Coronavirus Disease (COVID-19) Dashboard With Vaccination Data [Internet]. 2022. Available from:* [*https://covid19.who.int*](https://covid19.who.int)

*We are grateful for your acceptance to review our manuscript*.

Yours Sincerely,

Frederick Nchang Cho
