# Supplementary material for: Face mask ownership/utilisation and COVID-19 vaccine hesitancy amongst patients recovering from COVID-19 in Cameroon: A cross-sectional study
Source: PLoS One. 2023 Jan 20;18(1):e0280269. doi: 10.1371/journal.pone.0280269 (PMC9858007; doi:10.1371/journal.pone.0280269)
Supplement: Attachment — Submitted filename: Response to Reviewers.docx [file pone.0280269.s010.docx]

Infectious Disease Laboratory,

Faculty of Health Sciences,

University of Buea

Buea – Cameroon

Tuesday, 29 November 2022

The Academic Editor,

Plos One

Dear **Sir,**

**Response to Reviewers**

I am delighted once more to enclose herewith as requested, a revised version of manuscript: PONE-D-22-15424R2; **Face mask Ownership/Utilisation and COVID-19 Vaccine Hesitancy Amongst Patients Recovering from COVID-19 in Cameroon: A Cross-sectional Study**, for re-evaluation and possibly, acceptance for publication.

We have done our utmost best to objectively answer the questions and concerns raised.

Thus, we are submitting as requested, the following:

*A marked-up copy of manuscript:* ***Revised Manuscript with Track Changes****.*

*An unmarked version of revised manuscript without tracked changes:* ***Manuscript****.*

*A study information sheet.*

**CONCERNS RAISED BY REVIEWER #1**

***No more concerns were raised***.

**CONCERNS RAISED BY REVIEWER #2**

Line 206 and 207: Please check this out it is not possible for the number of persons (665) enrolled to be lower than the number households (2,126).

***We interviewed one person per household; only one who had suffered from COVID-19 and got treated. In the questionnaire, we enquired the number of persons living in the household (Question 7 on the questionnaire).***

***Thus, the statement is, 665 persons/household included in the study, 2,126 de facto household residents were counted. This implies that 2,126 household residents were counted in the 665 households. Thus, 1,461 (2,126 - 665) persons were exposed*.**

***Due to adjustments, the lines have changed to lines 203 – 204***.

**GENERAL CONCERNS**

The strength and limitation of study must present after discussion. So, please move its current place. Please explain more about limitation of the study to explain the potential bias in the study.

***We have moved the strength and limitation as requested.***

There is no study implication given. Please give in detail at the end.

***The conclusion has been modified to meet this demand; lines 415 – 423*.**

Please provide the response rate of the study.

***We were not very keen on the response rate; but a part is reported in the results section (Lines 202 - 203)*. *However, the response rate was 665/690*100 (92.9%), base on outliers*.**

Please provide study information sheet as supplementary file.

***This has been provided as requested.***

Please explain in details on comments given by reviewer. The peer reviewer are not satisficed with comments and does not explain well.

***We had previously explained all the comments and points raised by Reviewer number two; as*** ***Reviewer number one had validated the manuscript for publication.***

Please include your questionnaire inside the manuscript and explain all comments in manuscript in detail.

***We have included the questionnaire as demanded*. *The questionnaire had been submitted as and S1 Appendix; Survey Questionnaire (PDF) and S3 Appendix; Online version of questionnaire (***[***https://forms.gle/Wevui24dn5DEVE3A9***](https://forms.gle/Wevui24dn5DEVE3A9)***) in previous submissions*.**

Please provide strobe statement and strobe checklist as supplementary file.

***The STROBE checklist has been provided as requested*.**

Please provide raw data as well as supplementary file.

***The data sheet has been submitted as an excel sheet, together with earlier* *submitted S2 Appendix; Supplementary Tables (MS Word).***

***We are grateful for your acceptance to review our manuscript*.**

Yours Sincerely,

Frederick Nchang Cho
